# The proportion and effect of corticosteroid therapy in patients with COVID-19 infection: A systematic review and meta-analysis

**DOI:** 10.1371/journal.pone.0249481

**Published:** 2021-04-21

**Authors:** Junning Wang, Weixia Yang, Puwen Chen, Jianbin Guo, Rui Liu, Pengfei Wen, Kun Li, Yao Lu, Tao Ma, Xiaoli Li, Siqing Qin, Yumin Zhang, Yakang Wang

**Affiliations:** 1 Department of Respiratory, Honghui Hospital, Xi’an Jiaotong University, Xi’an, PR China; 2 Department of Pathology, Genertec Universal Xihang Hospital (Xi’an) Co., Ltd., Xi’an, PR China; 3 Department of Cardiology, The Second People’s Hospital of Foshan (Affiliated Foshan Hospital of Southern Medical University), Foshan, PR China; 4 Department of Orthopedics, Honghui Hospital, Xi’anJiaotong University, Xi’an, PR China; Ospedale Sant’Antonio, ITALY

## Abstract

**Objectives:**

Coronavirus disease 2019 (COVID-19) remains a global challenge. Corticosteroids constitute a group of anti-inflammatory and immunosuppressive drugs that are widely used in the treatment of COVID-19. Comprehensive reviews investigating the comparative proportion and efficacy of corticosteroid use are scarce. Therefore, we conducted a systematic review and meta-analysis of clinical trials to evaluate the proportion and efficacy of corticosteroid use for the treatment of COVID-19.

**Methods:**

We conducted a comprehensive literature review and meta-analysis of research articles, including observational studies and clinical trials, by searching the PubMed, EMBASE, Cochrane Controlled Trials Registry, and China Academic Journal Network Publishing databases. Patients treated between December 1, 2019, and January 1, 2021, were included. The outcome measures were the proportion of patients treated with corticosteroids, viral clearance and mortality. The effect size with the associated 95% confidence interval is reported as the weighted mean difference for continuous outcomes and the odds ratio for dichotomous outcomes.

**Results:**

Fifty-two trials involving 15710 patients were included. The meta-analysis demonstrated that the proportion of COVID-19 patients who received corticosteroids was significantly lower than that of patients who did not receive corticosteroids (35.19% vs. 64.49%). In addition, our meta-analysis demonstrated no significant difference in the proportions of severe and nonsevere cases treated with corticosteroids (27.91% vs. 20.91%). We also performed subgroup analyses stratified by whether patients stayed in the intensive care unit (ICU) and found that the proportion of patients who received corticosteroids was significantly higher among those who stayed in the ICU than among those who did not. The results of our meta-analysis indicate that corticosteroid treatment significantly delayed the viral clearance time. Finally, our meta-analysis demonstrated no significant difference in the use of corticosteroids for COVID-19 between patients who died and those who survived. This result indicates that mortality is not correlated with corticosteroid therapy.

**Conclusion:**

The proportion of COVID-19 patients who received corticosteroids was significantly lower than that of patients who did not receive corticosteroids. Corticosteroid use in subjects with severe acute respiratory syndrome coronavirus 2 infections delayed viral clearance and did not convincingly improve survival; therefore, corticosteroids should be used with extreme caution in the treatment of COVID-19.

## Introduction

Coronavirus disease 2019 (COVID-19) is a novel viral respiratory disease that surfaced in December 2019 and is caused by severe acute respiratory syndrome coronavirus 2 (SARS-CoV-2), a novel, highly diverse, enveloped, positive single-stranded betacoronavirus that belongs to the subgenus Sarbecovirus [1]. The rapid progression of the COVID-19 pandemic has become a global concern. By March 11, 2020, Central European Time, 114 countries had become involved, 118319 laboratory-confirmed infections had been reported, over 4000 deaths had occurred, and the World Health Organization (WHO) declared the COVID-19 outbreak a global pandemic [2]. By June 15, 2020, approximately 7823289 laboratory-confirmed cases had been identified worldwide, with 431541 deaths. Worryingly, the number of newly diagnosed patients continues to dramatically increase [3].

The clinical manifestations of COVID-19 in humans resemble those of viral pneumonia [4]. The pathogenesis of viral pneumonia may not be virus-induced cytopathy but rather an aberrant host immune reaction (e.g., cytokine storm) to the viral infection in all affected patients [5]. Because the immune pathogenesis of pneumonia may be the same in all infected patients, the timing of immunomodulator (corticosteroid) treatment is crucial, and the early control of initial immune-mediated lung injury is helpful for reducing patient morbidity and possible mortality [5]. Corticosteroids do not directly inhibit virus replication, and their main role is inhibiting inflammation and suppressing the immune response [6].

A wide range of variability in COVID-19 severity has been observed, ranging from asymptomatic to critical, and the symptoms of the disease are nonspecific, including self-reported fever, dry cough, fatigue, and myalgia with diarrhea. Severe cases of difficulty breathing, sepsis, and septic shock have been reported, progressing to a severe form of pneumonia in 10–15% of patients. Severe COVID-19 can lead to critical illness, with acute respiratory distress syndrome (ARDS) and multiorgan failure (MOF) as the primary complications, as well as fatal respiratory diseases [7]. Its epidemiological and clinical characteristics are slowly becoming evident. However, the pathogenic features of acute lung injury in COVID-19 and other infectious respiratory diseases remain unknown. Given the rapid emergence of COVID-19, currently, no pharmacological therapies with proven efficacy are available to treat this fatal disease [8]. Several companies have produced vaccines, but these vaccines are in phase 2 or 3 clinical trials, and the exact effect of these vaccines remains to be observed in the future [9]. SARS-CoV and Middle East respiratory syndrome coronavirus (MERS-CoV) share many genetic features; particularly, SARS-CoV-2 is highly homologous to SARS-CoV [10]. The phylogenetics and clinical features of COVID-19 resemble those of SARS and MERS; however, corticosteroid therapy in the latter two infections is controversial [11, 12]. The current guidance from the WHO regarding the clinical management of severe acute respiratory infection when SARS-CoV-2 infection is suspected (released: September 2, 2020) advises the use of systemic corticosteroids rather than no corticosteroids for the treatment of patients with severe and critical cases of COVID-19; however, the guidelines suggest not using corticosteroids in the treatment of patients with nonsevere cases of COVID-19 [13, 14]. Additionally, dexamethasone, which is a corticosteroid, has been found to improve survival in hospitalized patients who require supplemental oxygen, with the greatest effect observed in patients who required mechanical ventilation. Therefore, the use of dexamethasone is strongly recommended in this setting by the COVID-19 treatment Guidelines of the National Institutes of Health (last update: November 3, 2020) [15]. There have been several reports regarding the use of corticosteroids in addition to other therapeutics in patients with COVID-19, especially in persons with severe infection hospitalized in the intensive care unit (ICU); their impact on clinical outcomes remains highly controversial [8, 16, 17]. However, to date, data regarding the proportion and efficacy of corticosteroids in this setting are scarce [18, 19]. Understanding the evidence related to the efficacy and safety of corticosteroid treatment for COVID-19 is of immediate clinical importance. This meta-analysis aimed to evaluate the proportion and efficacy of the current options for the use of systemic corticosteroid therapy for COVID-19.

## Materials and methods

This systematic review and meta-analysis were conducted based on the Preferred Reporting Items for Systematic and Meta-analysis (PRISMA) protocols but were not registered in any registry.

### Search strategy

Two researchers (JN Wang and WX Yang) independently searched the PubMed, Embase, Cochrane Controlled Trials Registry, and China Academic Journal Network Publishing databases from December 1, 2019, to January 1, 2021, using the following key words: glucocorticoid or corticosteroid or adrenal cortex hormones or steroid or corticoid or corticoids or corticosteroids or glucocorticosteroid or glucocorticosteroids or methylprednisolone or budesonide or dexamethasone or Prednisone or prednisolone or methylprednisolone or hydrocortisone or cortisol. Each key word was searched with the following string of key words (using the “AND” operator): COVID-19 OR coronavirus OR "SARS-CoV-2" OR "novel coronavirus" OR 2019-nCoV OR "Severe Acute Respiratory Syndrome Coronavirus 2" OR "Corona Virus Disease 2019" OR COVID-19 OR COVID. No language restrictions were applied while searching for published studies.

### Inclusion and exclusion criteria

The inclusion criteria were as follows: 1) research articles, including observational studies and clinical trials, investigating the use of glucocorticoids in persons with COVID-19 infection who were diagnosed by real-time reverse transcription-polymerase chain reaction (RT-PCR) and underwent chest X-ray or chest computed tomography (CT) examination during hospitalization; (2) articles reporting outcomes regarding the proportion of glucocorticoids administered by severity and region, COVID-19 viral clearance and/or death; and (3) studies without restrictions based on the country in which the trial occurred and age.

The exclusion criteria were as follows: 1) studies involving patients post-transplantation or with a history of any organ transplantation; 2) studies that did not report original data, clear diagnostic criteria or data that could be summarized as the mean and standard deviation, and studies lacking reliable clinical data; and 3) conference abstracts or review articles.

Disagreements regarding the study selections were resolved by discussion with a review author (YK Wang) until consensus was reached.

### Data extraction

Two researchers (Yao Lu and JN Wang) independently performed the data extraction. The means were obtained from data tables or figures if no direct data were available in the article text or from the corresponding author. If the sample mean and standard deviation of the data could not be obtained from the authors, they were calculated from the sample size, median, range and/or interquartile range according to the procedures described in the articles by Wan X and Luo D et al [20, 21]. Disagreements regarding the data extraction were resolved by discussion with a review author (YK Wang) until a consensus was reached. The extracted data included the following: research type, author names, country, date of publication, sample size, number of patients treated with corticosteroids, dosage, duration and combination drugs, number of ICU admissions, invasive mechanical ventilation (IMV)/noninvasive ventilation (NIV), extracorporeal membrane oxygenation (ECMO), number of deaths, mortality, viral clearance time, comorbidity, classification, and length of in-hospital stay. The severity of COVID-19 was categorized as mild, common, severe or critical. The mild type was defined by mild symptoms, including any of the various signs and symptoms of COVID-19 (e.g., fever, cough, sore throat, malaise, headache, muscle pain, nausea, vomiting, diarrhea, and loss of taste and smell) without shortness of breath, dyspnea, or pneumonia on imaging. The common type was defined by respiratory tract symptoms and pneumonia on imaging. The severe type was characterized by dyspnea, respiratory rate ≥30/minute, blood oxygen saturation ≤93%, PaO2/FiO2 ratio <300, and/or >50% lung infiltration within 24–48 hours. The critical type was characterized by respiratory failure, septic shock, and/or multiple organ dysfunction/failure. We classified severe and critical cases as severe and common and mild cases as nonsevere. Finally, the data were imported into Review Manager 5.3 for the analysis.

### Assessment of study quality

Two researchers (PW Chen and JB Guo) independently assessed the quality of the included studies. The risk of bias was evaluated using the modified Jadad scale [22]. The following categories were included: “Was the study described as randomized?”, “Was the method used to generate the sequence of randomization described and appropriate (random numbers, computer-generated, etc.)?”, “Was the study described as double-blind?”, “Was the method of double-blinding described and appropriate (identical placebo, active placebo, dummy, etc.)?”, and “Was there a description of withdrawals and drop-outs?”. The Jadad scale is a five-point scale; a score of zero indicates poor quality evidence, and a score of five indicates high-quality evidence; therefore, trials with a score of 4 or 5 were considered to be of high methodological quality. Additionally, the Cochrane collaboration tool was used to address the risk of bias. Disagreements regarding the study quality were resolved by discussion with a review author (YK Wang) until a consensus was reached.

### Statistical analysis

The data were analyzed using the Cochrane Collaboration software Review Manager 5.3. The weighted mean difference (WMD) and the associated 95% confidence interval (CI) of the viral clearance as a continuous outcome were calculated, while the odds ratio (OR) and the associated 95% CI of dichotomous outcomes, including the proportion of cases treated with glucocorticoids and the mortality rate, were calculated.

Heterogeneity was assessed using an I^2^-test. A fixed-effects model was used to pool the data if there was no evidence of significant heterogeneity (I^2^≤50%). Otherwise, a random-effects model was used. Publication bias was assessed with funnel plots. The subgroup analyses were stratified by area (Wuhan, China; outside of Wuhan, China; and outside of China), severity (critical and severe), evidence grade age (pediatric or adult) and glucocorticoid dosage.

Ethics committee and/or institutional board approval were not required for this study.

## Results

### Trial characteristics

**Fig 1:**
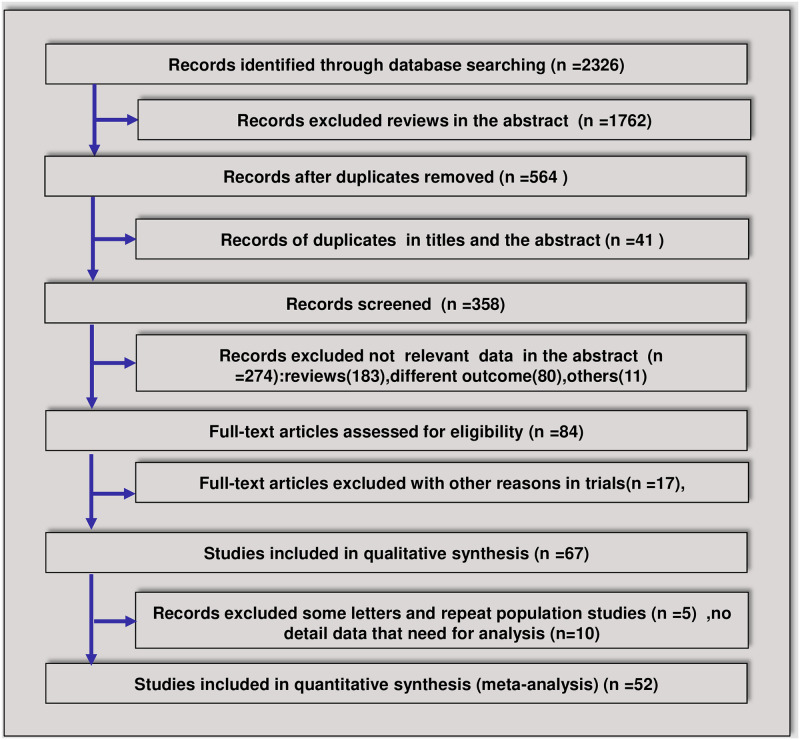
Flowchart of the article screening and selection process.

The searches identified 2326 relevant articles. Of these articles, 52 were eligible for inclusion according to our criteria for considering studies for this meta-analysis [18, 19, 23–72] (Fig 1). Forty-four trials were retrospective case series (RCS), and eight trials were randomized controlled trials (RCTs). In total, 11 RCT protocols were not included due to the lack of results (S1 File). In total, 15710 patients with COVID-19 were included in the analyses. Among the 52 included trials, 18 were multicenter trials, and 35 were single-center trials. Twenty-six trials were conducted in Wuhan, China, 17 trials were conducted outside of Wuhan, China, and 9 trials were conducted outside of China. In total, 12 studies performed analyses by severity; 4 trials divided the patients into ICU and non-ICU groups, and 8 trials divided the patients into severe or nonsevere groups. Viral clearance was compared in 5 trials. The effect on mortality was analyzed in 15 trials. Most trials indicated that 40–80 mg of methylprednisolone was used once or twice per day, ranging from 4–15 days. Antibiotics were not administered in three trials, 1 trial had no antibiotic-related data, and 51 trials administered antibiotics. In total, NIV was used in 2193 patients and IMV was used in 4729 patients in 27 trials to assist ventilation (S3 Fig). In total, 80 patients in 14 trials were treated with ECMO. Overall, 32 patients were included in Jacobs J et al’s article, and 4 of 5 survivors received steroids [29]. ECMO plays a role in the stabilization and survival of select critically ill patients with severe pulmonary and cardiac compromise; however, determining whether ECMO supplemented with corticosteroids is useful for improving the survival rate still requires more research. The most common complications were ARDS, acute coagulopathy, acute liver injury and acute kidney injury. The characteristics of the 52 included trials are summarized in Table 1.

**Table 1:** Characteristics of the studies included in the meta-analysis.

Study	Design	Age	Males (%)	Region	Site	Dose and duration	Combination drugs	Classification	Complications (%)	IMV/NIV	EC	ICU	Deaths	Hospitalization time	Follow up	Jadad scores
											MO					
Angus D [70] 2020	RCT	60	273 (71)	121 sites in 8 countries	Multiple centers	Hydrocortisone fixed 7 days (50 mg or 100 mg every 6 hours) and a shock-dependent course (50 mg every 6 hours)	Antiviral or immunoglobulin therapy and therapeutic anticoagulation	384 severe	Respiratory failure	213/114	3	384		21	NA	5
									ARDS							
									Heart failure							
									Shock							
Bai P [65] 2020	RCS	62.1 (30–92)	28 (48.3)	Wuhan, China	Single center	NA	Antibiotic and antiviral therapy	36 severe/22 critical	NA	7/NA		3	7 (28)	NA	NA	3
Bruno M [67] 2020.	RCT	60.1 ±15.8	187 (63.5)	Brazil	Multiple centers	Dexamethasone 20 mg/day, 5 days, 10 mg/day, 5 days or until ICU discharge	Hydroxychloroquine, azithromycin, other antibiotics, oseltamivir	moderate or severe	ARDS	187		187	176	28	NA	5
									Heart failure							
									Kidney failure							
Cao B [66] 2020	RCT	58.0 (49–68)	120 (60.3)	Shanghai, China	Single center	NA	Antibiotic and antiviral therapy	199 severe	Sepsis	32/29	4	NA	44 (28)	28	NA	5
									Respiratory failure							
									ARDS							
									Heart failure							
									Septic shock							
									Coagulopathy							
									Acute kidney injury							
Cao J [23] 2020	RCS	54.0 (37–67)	53 (52.0)	Wuhan, China	Single center	NA	Antiviral, antibiotic, and intravenous immunotherapy and Chinese medicine	NA	Shock 10 (9.8)	14/5	3	18	17 (15)	11 (7–15)	NA	3
									ARDS 20 (19.6)							
									Acute infection 17 (16.7)							
									Acute cardiac injury 15 (14.7)							
									Arrhythmia 18 (17.6)							
									Acute kidney injury 20 (19.6)							
									Acute liver injury 34 (33.3)							
Chen N [24] 2020	RCS	55.5 (21–82)	67 (68.0)	Wuhan, China	Single center	1–2 mg/kg/day; 3–15 days (median, 5 [3–7]).	Antibiotic, antifungal, antiviral and intravenous immunoglobulin therapy	2 severe	ARDS 17 (17)	4/13	3	23	11 (20)	NA	NA	3
									Acute renal injury 3 (3)							
									Acute respiratory injury 8 (8)							
									Septic shock 4 (4)							
									Ventilator-associated pneumonia 1 (1)							
Chen T [54] 2020	RCS	54 (20–91)	108 (53.2)	Wuhan, China	Single center	40–80 mg/day; 3–5 days	Expectorant, antiviral and immunoglobulin therapy	36 severe/34 critical	ARDS 18 (69.2)	39	NA		26 (40)	11 (1–45)	NA	3
									Sepsis/shock							
									Heart failure							
Deng Y [55] 2020	RCS	54.5 (33–74)	124 (55.1)	Wuhan, China	Two tertiary hospitals	NA	Antibiotics antifungal and immunoglobulin therapy	95 severe	ARDS 98 (89.9)	21/68	2	NA	109 (50)	8–16	NA	3
									Acute cardiac injury 65 (59.6)							
									Acute kidney injury 20 (18.3)							
									Shock 13 (11.9)							
									Disseminated intravascular							
									coagulation 7 (6.4)							
Dequin P [69] 2020	RCT	62.2	108 (69.8)	France	Multiple centers	Hydrocortisone 200 mg/d 7 days then decreased to 100 mg/d for 4 days and 50 mg/d for 3 days for a total of 14 days	Antibiotic, antiviral and immunoglobulin therapy	149 critical	ARDS	121/4	4	149	76	21	NA	5
									Sequential organ failure							
Ding Q [25] 2020	RCS	50.2 (39–66)	2 (40)	Wuhan, China	Cluster of cases	NA	Antibiotic, antiviral and transient hemostatic medication therapy	1 severe	ARDS 1 (20)	0/1	0	0	0	12–30	NA	1
									Acute liver injury 3 (60)							
									Diarrhea 2 (40)							
Du Y [26] 2020	RCS	65.8±14.2	62 (72.9)	Wuhan, China	Two hospitals	NA	Antibiotic, antifungal, antiviral, interferon and intravenous immunoglobulin therapy	60 mild/25 severe	Respiratory failure 80 (94.1)	18/61	0	NA	85 (37)	6.35 ± 4.51	36	3
									Shock 69 (81.2)							
									ARDS 63 (74.1)							
									Arrhythmia 51 (60)							
									Acute cardiac injury 38 (44.7)							
									Acute liver injury 30 (35.3)							
									Sepsis 28 (32.9)							
Emmi G [48] 2020	RCS	42 (36–48)	2 (15.4)	Tuscany, Italy	Single center	Prednisone equivalent (1.5–5) mg/day	Hydroxychloroquine, anti-rheumatic drugs and immunoglobulin therapy	1 severe	ARDS 1 (7.6)	NA	NA	1	NA	NA	14	3
Fang X [27] 2020	RCS	45.5 (24–74)	44 (56.4)	Anhui, China	Single center	Methylprednisolone 38–40 mg/day	Antiviral therapy, Chinese medicine	23 severe	ARDS 9 (11.5)	NA	NA	NA	NA	NA	NA	3
Gao T [38] 2020	RCS	41.0±16.4	19 (47.5)	Xianyang, Liancheng, China	Two hospitals	Methylprednisolone 40~80 mg/time, twice/day	Antibiotic, antifungal, antiviral and immunoglobulin therapy	3 mild/36 common/1 severe	NA	NA	NA	NA	0	NA	NA	3
G uan W [46] 2020	RCS	47.0 (35–58)	640 (58.2)	Mainland, China	552 hospitals in 30 provinces	NA	Antibiotic, antifungal, antiviral and immunoglobulin therapy	52 severe	Septic shock 12 (1.1)	25/56	5	55	15 (15)	12.0 (10.0–14.0)	NA	3
									ARDS 37 (3.4)							
									Acute kidney injury 6 (0.5)							
									Disseminated intravascular							
									coagulation 1 (0.1)							
Guo T [44] 2020	RCS	58.5 ±14.66	91 (48.7)	Wuhan, China	Single center	Methylprednisolone 40–80 mg every day	Antiviral, antibiotic, immune glucocorticoid therapy	NA	ARDS 46 (24.6)	45	NA	NA	43 (30)	16.63 ± 8.12	NA	3
									Acute coagulopathy 42 (34.1)							
									Acute liver injury 19 (15.4)							
									Acute kidney injury 18 (14.6)							
Han Y [28] 2020	RCS	57.3 (44–81)	1 (33.3)	Wuhan, Yiyang, China	Familial cluster	Prednisone 7.5 mg/d and steroids 80 mg/d	Antibiotic, antiviral, interferon and intravenous immunoglobulin therapy	1 severe	NA	NA	NA	1	NA	NA	NA	1
Hong K [47] 2020	RCS	55.4±17.1	38 (38.8)	Daegu, South Korea	Single center	Methylprednisolone 40–80 mg every day	Antibiotic, antiviral and immunoglobulin therapy	NA	ARDS 18 (18.4)	NA	NA	13	5	NA	NA	3
									Septic shock 9 (9.2)							
									Acute cardiac injury 11 (11.2)							
									Acute kidney injury 9 (9.2)							
Huang C [62] 2020	RCS	49 (41–58)	30 (73)	Wuhan, China	Single center	Methylprednisolone 40–120 mg per day	Antibiotic, antiviral and immunoglobulin therapy	NA	ARDS 12 (29)	2/10	2		6 (17)	NA	NA	4
									Acute cardiac injury 5 (12)							
									Acute kidney injury 3 (7)							
									Secondary infection 4 (10)							
									Shock 3 (7)							
Huang Q [61] 2020	RCS	41 (31–51)	28 (51.9)	Wuhan, China	Four hospitals	NA	Antibiotic, antiviral and immunoglobulin therapy	51 common/3 severe	ARDS 3 (5.6)	NA	NA	NA	0	9 (7–12)	NA	3
Jeronimo C [68] 2020	RCT	55±15	254 (64.6)	Manaus, Brazil	Single center	Methylprednisolone 0.5 mg/kg bid, 5 days	Antibiotic and antiviral therapy	393 severe or critical	NA	133/138		126	148	28	NA	5
Jacobs J [29] 2020	RCS	52 (12–49)	22 (68.8)	Unites States	9 different hospitals	NA	Antibiotic, antiviral and immunoglobulin therapy	32 critical	Pulmonary failure	NA	32		10 (23)	24	NA	3
									Heart failure							
									ARDS							
Li Q [71] 2020	RCS	59 (43–70)	258 (54.3)	Shanghai, China	19 different hospitals	Methylprednisolone 20–40 mg/day, 3–5 days	Antibiotic, antifungal, antiviral and immunoglobulin therapy	475 nonsevere	NA	NA	NA	NA	1	50	NA	3
Li R [30] 2020	RCS	50 ± 14	120 (53.3)	Wuhan, China	Single center	NA	Antibiotic, antiviral and immunoglobulin therapy	37 severe	NA	NA	NA	NA	NA	NA	NA	3
Li X [31] 2020	RCS	60 (48–69)	279 (50.9)	Wuhan, China	Single center	Prednisone cumulative dose, 200 (0–450) mg, 4 days	Antibiotic, antiviral and immunoglobulin therapy	153 severe	ARDS 210 (38.3)	25/78	NA	NA	90 (37)	NA	38	3
									Cardiac injury 119 (21.7)							
									Liver dysfunction 106 (19.3)							
									Acute kidney injury 95 (17.3)							
									Bacteremia 42 (7.7)							
Liu J [40] 2020	RCS	64 (54–73)	452 (58.4)	China	Multiple centers	NA	Antibiotic, antiviral and immunoglobulin therapy	774 severe	ARDS	119/157	7	277	290	32	NA	3
									Myocardial							
									Liver injury							
									Shock							
Lian J [32] 2020	RCS	41.15±11.38 (<60)	407 (51.6)	Wuhan, China	Single center	Methylprednisolone 40–80 mg/daily, 15 days	Antibiotic, antiviral and immunoglobulin therapy	710 mild/61 severe/17 critical	ARDS 58	11/7	0	86	0	NA	NA	3
									Septic shock 2							
									Abnormal liver function 82							
		68.28±7.314 (≥60)							Acute kidney injury 13							
Lian JS [56] 2020	RCS	45 (5–88)	243 (52.3)	Zhejiang, China	Multiple centers	Methylprednisolone 40 (40–80), 7 days	Antibiotic, antiviral and immunoglobulin therapy	20 mild/ 396 common)	ARDS 11 (2.37)	4/4	0	4	0	NA	NA	3
								41severe/critical	Shock 1 (0.22)							
									Liver injury 61 (13.12)							
Ling Y [33] 2020	RCS	44.0 (34–62)	28 (42.4)	Shanghai, China	Single center	NA	NA	NA	NA	NA	NA	NA	NA	NA	14	3
Luo P [50] 2020	RCS	73 (62–80)	12 (80.0)	Wuhan, China	Single center	Methylprednisolone 40 mg bid	Tocilizumab treatment	2 common /6 severe/7 critical	NA	NA	NA	NA	3 (38)	NA	NA	3
Mo P [34] 2020	RCS	54 (42–66)	86 (55.5)	Wuhan, China	Single center	NA	Antibiotic, antiviral, interferon and intravenous immunoglobulin therapy	63 common /55sever/37critical	Severe pneumonia	36	NA	NA	22 (40)	10	50	3
									Pulmonary edema							
									ARDS							
									Multiple organ failure							
Ni Q [39] 2020	RCS	52 (45–62)	29 (56.9)	Zhejiang, China	Single center	Methylprednisolone 0.75~1.50 mg/d	Antibiotic, antiviral, and immunoglobulin therapy	13 common	NA	NA	NA	NA	NA	NA	NA	3
								26severe/12critical								
Pang X [37] 2020	RCS	45. 1 (5–91)	45 (57)	Anhui, China	Single center	1–2 mg/kg/d, more than 5 days	Antibiotic, antiviral, and immunoglobulin therapy	55 common /21 severe/3 critical	Severe pneumonia	1	0	1	1 (27)	NA		3
									Pulmonary edema							
									ARDS							
									Multiple organ failure							
Peter H [72] 2020	RCT	66.1	4112 (64)	United Kingdom	Multiple centers	Dexamethasone 6 mg/day, 10 days	Antibiotic, antiviral, and immunoglobulin therapy	NA	NA	1007/3883	NA	NA	1147	28	28	5
Petersen M [49] 2020	RCT	57 (52–75)	23 (79)	Denmark	Multiple centers	Hydrocortisone 200 mg/d, 7 days	Antibiotic, antiviral, and immunoglobulin therapy		Sepsis	15	NA	NA	8	28	365	5
									Shock							
									Fungal							
									Infection							
Qiu C [57] 2020	RCS	43 (8–84)	49 (47.1)	Wuhan, China	Single center	NA	Antibiotic, antiviral, and immunoglobulin therapy	16 severe	ARDS 12 (11.54)	3/4	NA	9	1 (32)	10.45±3.79	33	3
									Acute kidney injury 2 (1.92)							
									Abnormal liver function 5 (4.81)							
									Cardiac injury 3 (2.14)							
									Shock 2 (1.92)							
Shen Q [41] 2020 (children)	RCS	7.6 (1–12)	3 (25)	Changsha, China	Single center	NA	Antibiotic, antiviral, and immunoglobulin therapy	0	0	0	0	0	0	12–16	49	1
Sun L [51] 2020	RCS	44.0 (34–56)	31 (56.4)	Beijing, China	Single center	40–80 mg/day	Antibiotic, antiviral, interferon, and immunoglobulin therapy and Chinese medicine	40 mild/ common, 15 severe/ critical	Abnormal liver and kidney function	3/5	0	0	0	NA		3
Wan S [42] 2020	RCS	47 (36–55)	72 (53.3)	Chongqing, China	Single center	NA	Antibiotic, antiviral, and immunoglobulin therapy and Chinese medicine	95 mild /40 severe	ARDS 21 (15.6)	34/1	0	0	1 (16)	NA	NA	3
									Acute cardiac injury 10 (7.4)							
									Acute kidney injury 5 (3.7)							
									Secondary infection 7 (17.5)							
									Shock 1 (0.7)							
Wang D [35] 2020	RCS	56 (22–92)	75 (54.3)	Wuhan, China	Single center	NA	Oseltamivir and antibacterial therapy	NA	ARDS	17/15	4	26	6 (27)	NA	34	3
Wang L [58] 2020	RCS	42 (34–53)	11 (42.3)	Shandong, China	Single center	NA	Antibiotic, antiviral, and immunoglobulin therapy, Chinese medicine, and gastric mucosal protection	NA	NA	NA	NA	NA	NA	NA	NA	3
Wang Y [18] 2020	RCS	54 (48, 64)	26 (57)	Wuhan, China	Single center	Methylprednisolone 1–2 mg/kg/day for 5–7 days	Antibiotic, antiviral and immunoglobulin therapy	46 severe	NA	7/3	0	46	3 (36)	NA	NA	3
Wang YM [52] 2020	RCT	65 (56–71)	89 (56)	Hubei, China	Ten hospitals	8 days	Antibiotic, antiviral, interferon, vasopressor and immunoglobulin therapy	NA	ARDS 22	21/17	2	NA	32 (35)	8 (6–9) vs 15 (9–19)	64	5
									Pulmonary embolism 2							
									Cardiac arrest 1							
									Septic shock 2							
Wu C [19] 2020	RCS	51 (43–60)	128 (63.7)	Wuhan, China	Single center	NA	Antibiotic, antiviral, interferon, antioxidant and immunoglobulin therapy	NA	ARDS 84 (41.8)	61/5	1	53	44 (32)	13 (10–16)	50	3
Xu K [53] 2020	RCS	52 (43, 63)	66 (58.4)	Wuhan, China	Single center	Methylprednisolone 0.5–1 mg/kg	Antiviral, interferon and immunoglobulin therapy	32 severe/23 critical	ARDS 23	0/18				21	NA	3
Yang W [59] 2020	RS	45.11 ± 13.35	81 (54.4)	Zhejiang, China	Single center	NA	Antibiotic, antiviral, interferon and immunoglobulin therapy	NA	NA	2/0	0	0	0	NA	29	3
Yang X [64] 2020	RCS	59 ±13	35 (67)	Wuhan, China	Single center	NA	Antibiotic, antiviral, vasoconstrictive and immunoglobulin therapy	52 critical	ARDS 35 (67)	22/29	6	52	32 (26)	NA	NA	3
									Acute kidney injury 15 (29)							
									Cardiac injury 12 (23)							
									Liver dysfunction 15 (29)							
									Pneumothorax 1 (2)							
Zha L [36] 2020	RCS	39 (32–54)	20 (64%)	Wuhu, Anhui province, China	Two designated hospitals	Methylprednisolone 40 mg once or twice per day	Antibiotics, moxifloxacin, lopinavir/ritonavir and interferon alfa; umifenovir, lopinavir/ritonavir and interferon alfa	NA	Liver injury 12 (39)	NA	NA	NA	0	18.5 (16–21)	NA	3
						5 days (iqr, 4.5–5.0 days)										
Zhang Y [43] 2020	RCS	62.7±14.2	85 (51.2)	Wuhan, China	Single center	Methylprednisolone 1–2 mg/kg/d, 3–7 days	Antibiotic, antiviral, and intravenous immunoglobulin therapy and tocilizumab	100 severe/36 critical	Acute kidney injury	22/11		7	24 (41)	23.0±12.2	NA	3
									Cardiac injury							
Zhao X [60] 2020	RCS	46.00	49 (53.8)	Jingzhou, China	Single center	NA	Antibiotic, antiviral and immunoglobulin therapy	61 mild/30 severe	Cardiovascular jury 14 (15.4)	5	NA	NA	2 (25)	NA	25	3
									Digestive tract jury 14 (15.4)							
									Liver jury 18 (19.8)							
									Renal jury 5 (5.5)							
									Coagulation dysfunction 19 (20.9)							
Zheng F [45] 2020	RCS	3 (2–9)	14 (56.0)	Hubei, China	10 hospitals	2 mg/kg/day	Antibiotic, antiviral and immunoglobulin therapy	15 mild/2 critical	ARDS	2	0	2	0	NA	NA	3
Zhou F [63] 2020	RCS	56 (46–67)	119 (62)	Wuhan, China	2 hospitals	NA	Antibiotic, antiviral and immunoglobulin therapy	66 severe/53 critical	Sepsis 112 (59)	32/6	3	50	54 (33)	11 (7–14)	NA	3
									Respiratory failure 103 (54)							
									ARDS 59 (31)							
									Heart failure 44 (23)							
									Septic shock 38 (20)							
									Coagulopathy 37 (19)							
									Acute cardiac injury 33 (17)							
									Acute kidney injury 28 (15)							

ARDS, acute respiratory distress syndrome; ECMO, extracorporeal membrane oxygenation; ICU, intensive care unit; IMV, invasive mechanical ventilation; NIV, noninvasive ventilation; RCS, retrospective case series; RCT, randomized controlled trial. Age (median/mean [range/IQR], years); length of in-hospital stay (median/mean [range/IQR].

### Proportion of corticosteroid treatments

**Fig 2:**
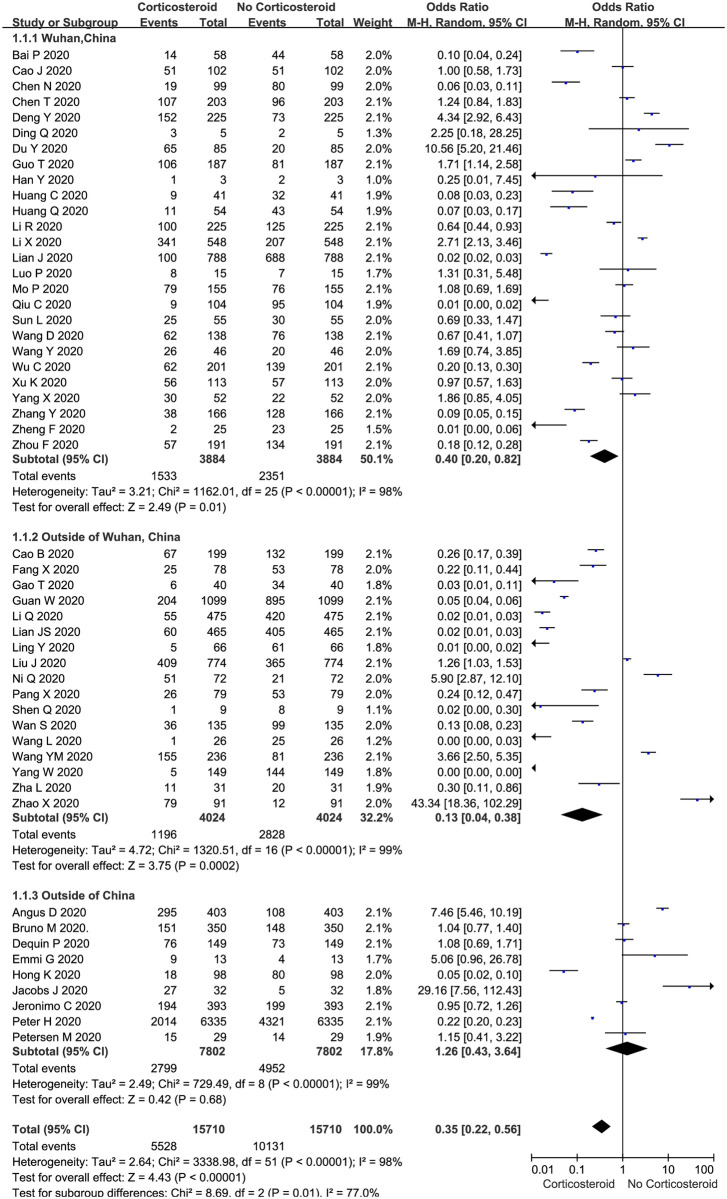
Proportion of corticosteroid treatments in COVID-19 patients: Overall and subgroup analyses stratified by region.

The proportion of COVID-19 patients treated with corticosteroids compared to those who were not was described in all 52 included trials (n = 15710 patients). The meta-analysis demonstrated that the proportion of COVID-19 patients treated with corticosteroids was significantly lower than that of patients who were not treated with corticosteroids (35.19% vs. 64.49%, 5528 vs. 10131 OR: 0.35, 95% CI: 0.22–0.56, *P* <0.01; Fig 2) in both adult and pediatric cases (S4A Fig). There was evidence of significant heterogeneity among the trials (*P* <0.01, *I*^*2*^ = 98%). There was no significant difference between the patients who were treated with corticosteroids and those not treated with corticosteroids among those with low and high Jadad scores (S4B Fig).

### Comparison of the proportion of severe and nonsevere cases treated with corticosteroids

**Fig 3:**
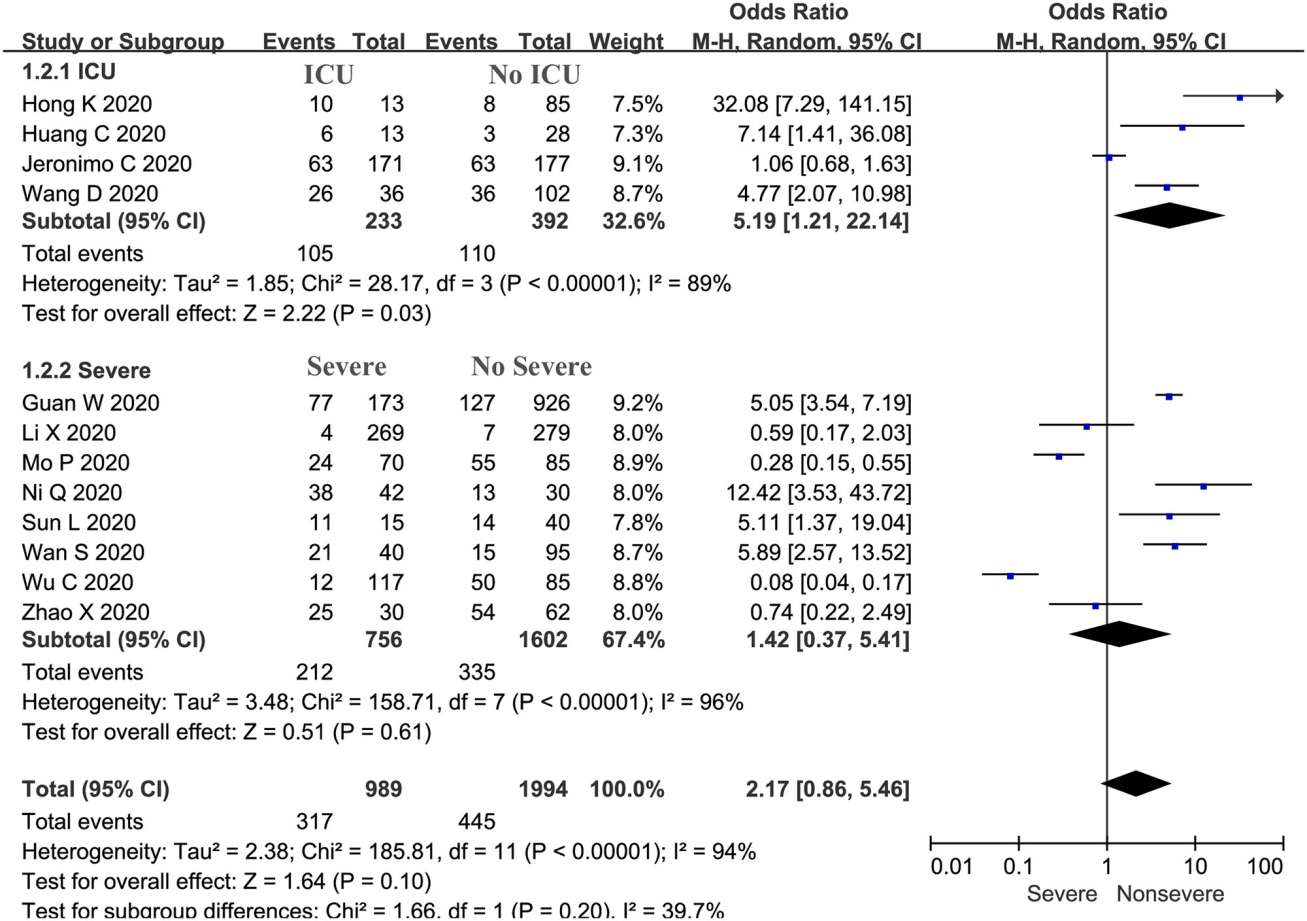
Proportions of severe and nonsevere cases treated with corticosteroids: Overall and subgroup analyses stratified by severity.

The proportion of severe cases treated with corticosteroids was 32.05% (n = 317), while 22.31% (n = 445) of nonsevere cases were treated with corticosteroids in 12 trials (n = 2983 patients). Our meta-analysis demonstrated a significant difference in the proportions of severe plus ICU and nonsevere plus no ICU cases treated with corticosteroids (OR: 2.17, 95% CI: 0.86–5.46, *P* = 0.04; Fig 3). There was evidence of significant heterogeneity among the trials (*P* <0.01, *I*^*2*^ = 94%).

### Effect of corticosteroid use on viral clearance

**Fig 4:**
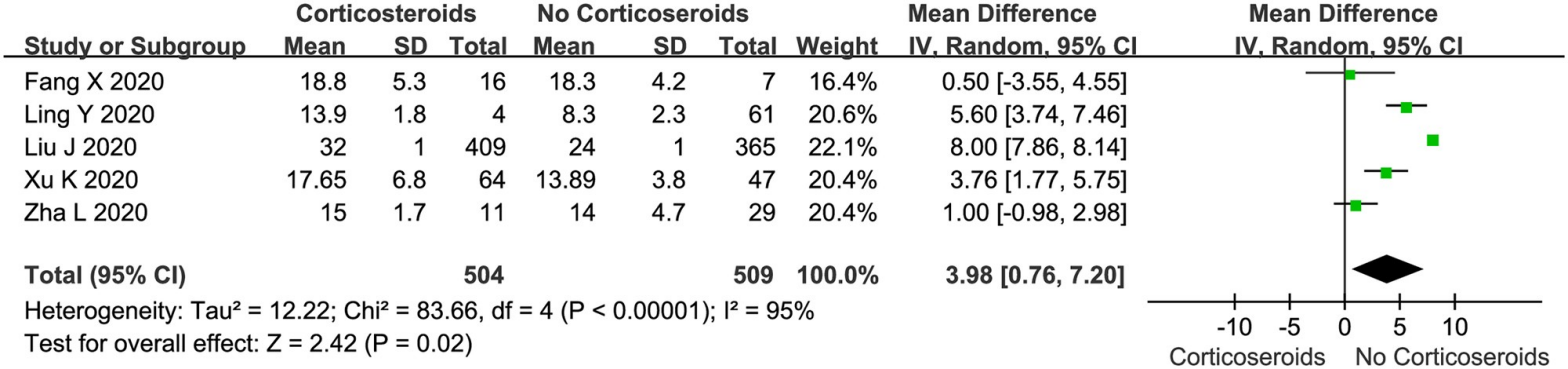
Corticosteroid vs. no corticosteroid treatment: Viral clearance time (days).

We evaluated the viral clearance time in patients treated with corticosteroids compared with that in patients who were not treated with corticosteroids using a random-effects model (Fig 4). Five studies reported the outcome of viral clearance. In all 5 studies, viral clearance was confirmed by serial RT-PCR of samples from throat swabs or sputum; in the 5 studies, clearance was defined as at least two consecutive negative results. The pooled estimates showed that corticosteroid treatment significantly delayed the viral clearance time (WMD: 3.98, 95% CI: 0.76–7.02, *P* < 0.05; *I*^*2*^ = 95%). However, there was significant heterogeneity among the studies.

### Effect of corticosteroid use on mortality

**Fig 5:**
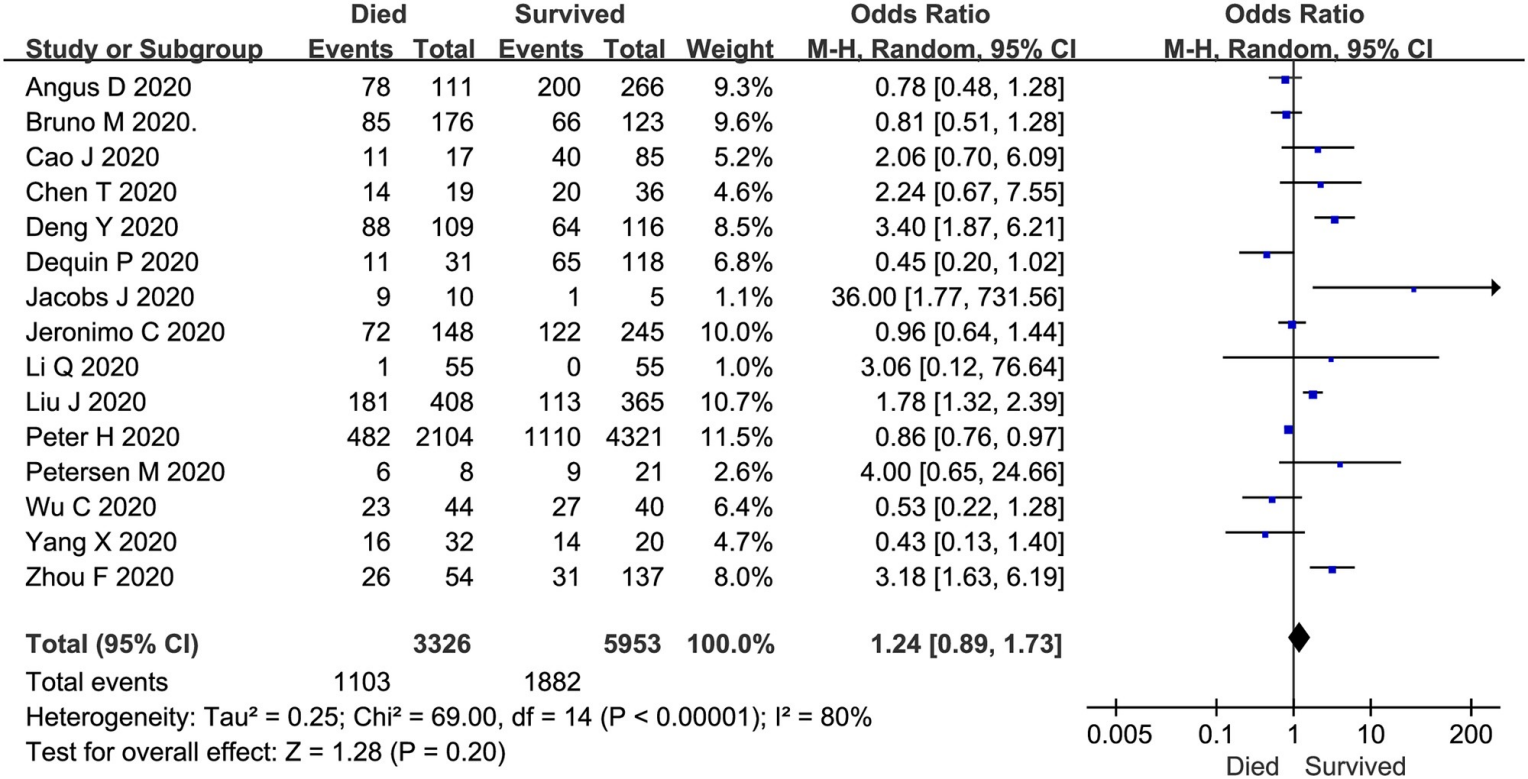
Corticosteroid vs. no corticosteroid treatment: Mortality of studied subjects (both groups received corticosteroids).

The mortality of COVID-19 patients treated with corticosteroids for 4–15 days was described in 15 trials (n = 9279 patients). The meta-analysis demonstrated no significant difference in the use of corticosteroids between COVID-19 patients who died and those who survived (overall OR: 1.24, 95% CI 0.89–1.73, *P* = 0.2; Fig 5). There was evidence of significant heterogeneity among the trials (*P* < 0.01, *I*^*2*^ = 80%).

### Subgroup and sensitivity analyses

The subgroup analyses stratified by region indicated that the proportion of COVID-19 patients treated with corticosteroids was significantly lower than that of patients who were not treated with corticosteroids in Wuhan, China (OR: 0.40, 95% CI: 0.20–0.82, *P* = 0.01; *I*^*2*^ = 98%, Fig 2) and outside of Wuhan (OR: 0.13, 95% CI: 0.04–0.38, *P* < 0.01; *I*^*2*^ = 99%, Fig 2), but there was no significant difference outside of China (OR: 1.26, 95% CI: 0.43–3.64, *P* = 0.68; *I*^*2*^ = 99%, Fig 2).

The subgroup analyses were also stratified by whether patients stayed in the ICU and by severity. Patients who were identified as having severe or critical disease were collectively included in the “severe” group, while those with mild and common COVID-19 were included in the “nonsevere” group. The subgroup analysis indicated that the proportion of patients treated with corticosteroids among ICU patients was significantly higher than that among non-ICU patients (OR: 5.19, 95% CI: 1.21–22.14, *P* = 0.03; *I*^*2*^ = 89%; Fig 3), but there was no significant difference in the proportion of patients with critical or severe disease and mild or common disease treated with corticosteroids (OR: 1.42, 95% CI: 0.37–5.41, *P* = 0.61; *I*^*2*^ = 96%; Fig 3).

The subgroup analyses were also stratified by the dosage of corticosteroids and whether the patients were ventilated. The main dosage of corticosteroids used was 40–80 mg/day. The number of patients treated with corticosteroids at 40–80 mg/day was significantly lower than the number of patients not treated with corticosteroids (557 vs. 1580; S2 Fig); however, there were no significant differences in the number of patients treated by weight, treated with less than 40 mg/day, treated with more than 80 mg/day, and not treated with corticosteroids. There was also no significant difference in the number of ventilated and nonventilated patients (2193 vs. 4729; S3 Fig).

### Assessment of study quality

The level of evidence in each trial was graded from 1 to 5 according to the Jadad quality score (Table 1 and S2 Table). Regarding publication bias, the shape of the funnel plot showed obvious asymmetry for trials investigating the proportion of corticosteroid use in COVID-19 patients regardless of region or severity (S1A and S1B Fig) and slight asymmetry for trials investigating the effect on viral clearance (S1C Fig) and mortality (S1D Fig).

Additionally, the risk of bias, as assessed by the Cochrane tool, is summarized in S5 Fig and presented in detail in S6 Fig. The main limitations of the included trials were selection bias and performance bias because most studies were not randomized or blinded.

## Discussion

Since the outbreak of the novel SARS-CoV-2 infection, no effective antiviral treatment has been developed. COVID-19 patients are mainly treated with symptomatic therapy. In clinical practice, corticosteroids are widely used in the symptomatic treatment of severe viral pneumonia. However, whether COVID-19 patients should be adjunctively treated with corticosteroids remains highly controversial. The main pathological feature of COVID-19 pneumonia is an inflammatory reaction accompanied by deep airway and alveolar destruction [73]. The current hypothesis is that lung injury is not associated with direct virus-induced injury but that COVID-19 invasion triggers immune and inflammatory responses that lead to the activation of immune cells (macrophages, T and B lymphocytes, granulocytes, and monocytes) to release numerous pro- and anti-inflammatory cytokines, including TNF-α, IL-1β, and IL-6, and markedly increased levels of inflammatory markers, such as C-reactive protein and the erythrocyte sedimentation rate [74]. The overwhelming secretion of cytokines causes severe alveolar and deep airway damage, which manifests as extensive damage to pulmonary vascular endothelial and alveolar epithelial cells and increased pulmonary vascular permeability, resulting in pulmonary edema and hyaline membrane formation [75]. Lung histological examinations have shown diffuse alveolar damage with cellular fibromyxoid exudate and hyaline membrane formation, which resembles ARDS [73]. Further autopsy revealed bilateral diffuse alveolar injury with fibrous mucinous exudate and interstitial mononuclear inflammatory infiltration dominated by lymphocytes, which is very similar to SARS-CoV and MERS-CoV infections [73]. This finding indicates that COVID-19 infection is usually accompanied by increased immune and inflammatory responses and that the concentrations of immune factors are associated with the severity of the disease [62]. Corticosteroids are classical immunosuppressive drugs that perform key physiological processes, including exerting inhibitory effects on the immune response and playing anti-inflammatory roles to reduce systemic inflammation [16, 76]. Both aspects are important for stopping or delaying the progression of pneumonia. Low-dose corticosteroids have been proven to be effective in the treatment of viral pneumonia due to their excellent pharmacological effects on the suppression of the immune system to prevent the development of related autoimmune diseases and dysfunctional systematic inflammation [77].

In this meta-analysis, the proportion of COVID-19 patients treated with corticosteroids was significantly lower than that of patients who did not receive corticosteroids. The subgroup analyses stratified by region showed that the proportion of COVID-19 patients treated with corticosteroids was significantly lower than that of patients who were not in Wuhan, China, outside of Wuhan, and outside of China. The results of this study indicate that the clinical application of corticosteroids is not very common. Thus, the use of corticosteroids could be regarded as a double-edged sword [16].

Studies have indicated that patients with severe disease are more likely to require adjunctive corticosteroid therapy [77]. However, our meta-analysis demonstrated no significant difference in the proportion of severe and nonsevere cases treated with corticosteroids. This finding differs from the results of previous research. We speculate that the reason underlying this inconsistency is an unsuitable population selection as follows: patients with mild or common COVID-19 might not be included in a target population to assess the efficacy of corticosteroids in most studies. We also performed subgroup analyses stratified by severity, which indicated that the proportion of corticosteroid use in ICU patients was significantly higher than that in non-ICU patients. These results indicate that ICU patients were more likely to require corticosteroid therapy. The meta-analysis by Li Huan et al. reported that evidence suggests that ICU inpatients with coronavirus infections were more likely to receive corticosteroids than non-ICU inpatients [78].

The results of our meta-analysis indicate that corticosteroid treatment significantly delayed the viral clearance time. A study by Russell D.C. et al showed a delay in viral RNA clearance from the respiratory tract and suggested that this delay followed corticosteroid treatment for MERS-CoV infection [14]. Moreover, a prospective, randomized, double-blinded, placebo-controlled trial investigating SARS compared early adjunctive hydrocortisone treatment (before day seven of the illness) with a placebo and showed that early adjunctive hydrocortisone therapy in patients was associated with delayed SARS-CoV RNA clearance in plasma [79].

The meta-analysis demonstrated no significant difference in the use of corticosteroids between COVID-19 patients who died and those who survived. These results indicate that mortality is not correlated with corticosteroid therapy; there was no favorable impact on the endpoint of death. In a retrospective cohort study involving 309 patients who were critically ill with MERS [12], the authors reported that there was no difference in 90-day mortality between patients treated with corticosteroids and those not treated with corticosteroids, but the corticosteroid treatment was associated with delayed MERS-CoV RNA clearance from respiratory tract secretions. This finding was somewhat supported by our systematic review. Glucocorticoid therapy was associated with delayed SARS-CoV-2 RNA clearance after adjusting for baseline and time-varying confounding factors [33]. However, the WHO’s rapid evidence appraisal of COVID-19 therapies by a working group conducting a prospective meta-analysis showed that in clinical trials of patients critically ill with COVID-19, compared with usual care or placebo, the administration of systemic corticosteroids was associated with a lower 28-day all-cause mortality rate [80], which differs from our results because we included mild, common and severe cases in our meta-analysis.

There are some limitations to this meta-analysis. First, some included studies were early retrospective cohort studies with small patient sample sizes and historical control studies of this emerging pathogen, and we found substantial heterogeneity among studies with a low level of evidence, which restricted the quality grade of the effects. Larger-scale RCTs are urgently needed. Second, there is no uniform standard for the dosage or initiation time of the administration of the corticosteroid regimens used in the different studies. For instance, in future research, corticosteroids should be used at an early stage of the illness. Third, antiviral agents might be confounders to corticosteroid use and their effects. Other co-treatments might have influenced our results. Fourth, our study was not registered, and the study populations only included hospitalized patients. Finally, due to the ongoing outbreak of COVID-19, many regions affected by COVID-19 have not published results in their populations, which may lead to publication bias.

## Conclusions

The proportion of COVID-19 patients treated with corticosteroids was significantly lower than that of patients who were not treated with corticosteroids. The subgroup analyses stratified by severity indicated that the proportion of corticosteroid use in ICU patients was significantly higher than that in non-ICU patients. Corticosteroid use in subjects with SARS-CoV-2 infection resulted in delayed viral clearance and did not convincingly improve survival in all patients. Therefore, corticosteroids should be used with extreme caution in the treatment of COVID-19. Nevertheless, further multicenter, larger, randomized, controlled clinical trials are needed to verify this conclusion.

## Supporting information

S1 Fig A. Funnel plot of the proportion of corticosteroid treatments in COVID-19 patients by region. B. Funnel plot of the proportion of corticosteroid treatments in COVID-19 patients by severity. C. Funnel plot of the effect of corticosteroid treatments on viral clearance in COVID-19 patients. D. Funnel plot of mortality.
(PDF)

S2 Fig Proportion of corticosteroid treatments in COVID-19 patients: Overall and subgroup analyses stratified by dosage. (PDF)

S3 Fig Proportion of corticosteroid treatments in COVID-19 patients: Analyses stratified by ventilation. (PDF)

S4 Fig Proportion of corticosteroid treatments in COVID-19 patients: Overall and subgroup analyses stratified by risk of bias (B) and age (A). (PDF)

S5 Fig Risk of bias of studies included in the meta-analysis presented as percentages using the Cochrane tool. (PDF)

S6 Fig Summary of the risk of bias of each included study by risk of bias item. (PDF)

S1 Table PRISMA 2009 checklist. (DOC)

S2 Table Jadad quality scores. (DOCX)

S1 File 11 RCT protocols without results. (DOCX)

S1 Data (XLSM)

S2 Data (CSV)

## Abbreviations

ARDS: acute respiratory distress syndrome
CI: confidence interval
COVID-19: coronavirus disease 2019
ICU: intensive care unit
MOF: multiorgan failure
OR: odds ratio
RCTs: randomized controlled trials
WMD: weighted mean difference
