# Supplementary figures and images for: The proportion and effect of corticosteroid therapy in patients with COVID-19 infection: A systematic review and meta-analysis
Source: PLoS One. 2021 Apr 21;16(4):e0249481. doi: 10.1371/journal.pone.0249481 (PMC8059814; doi:10.1371/journal.pone.0249481)

**A**

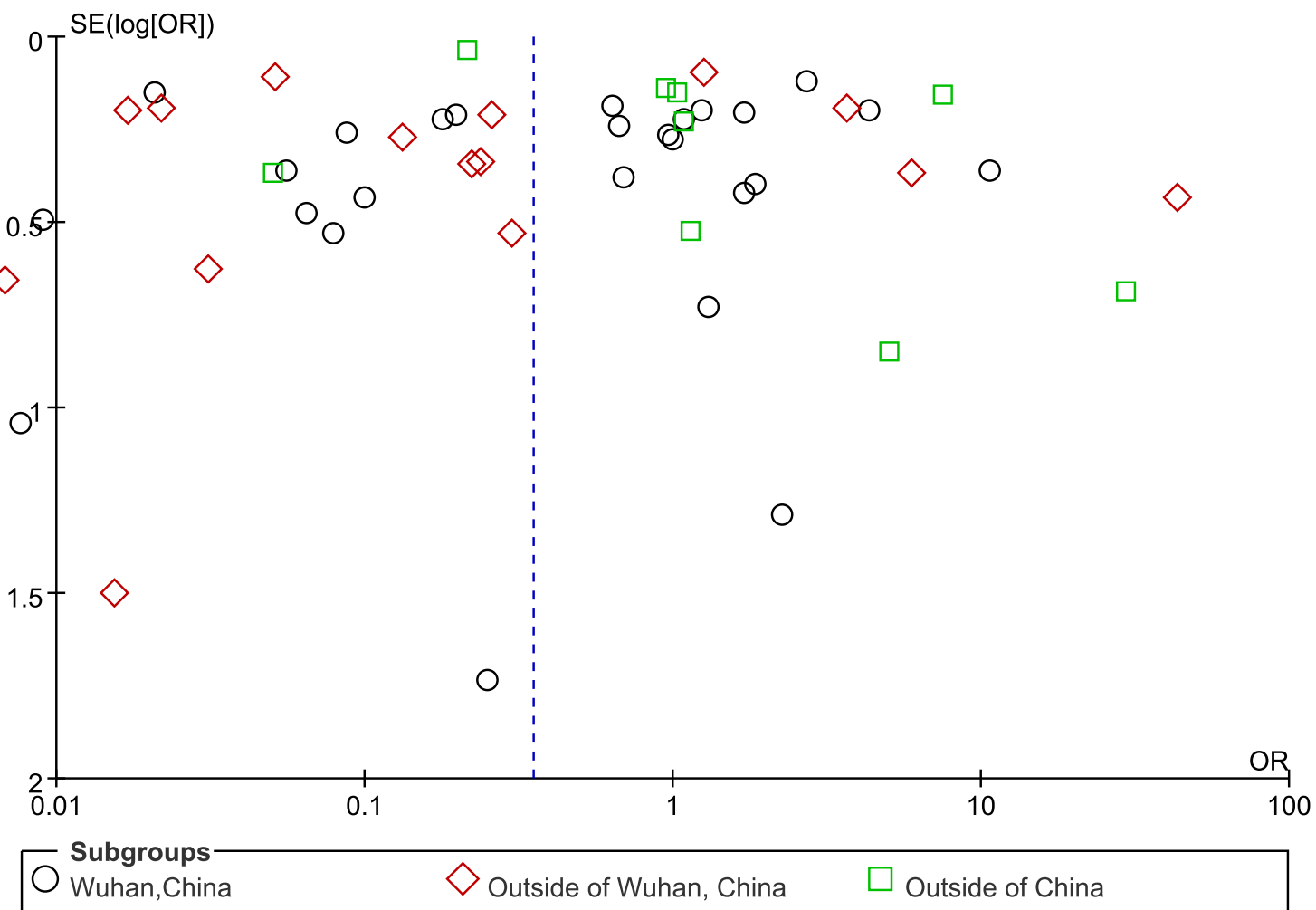

B

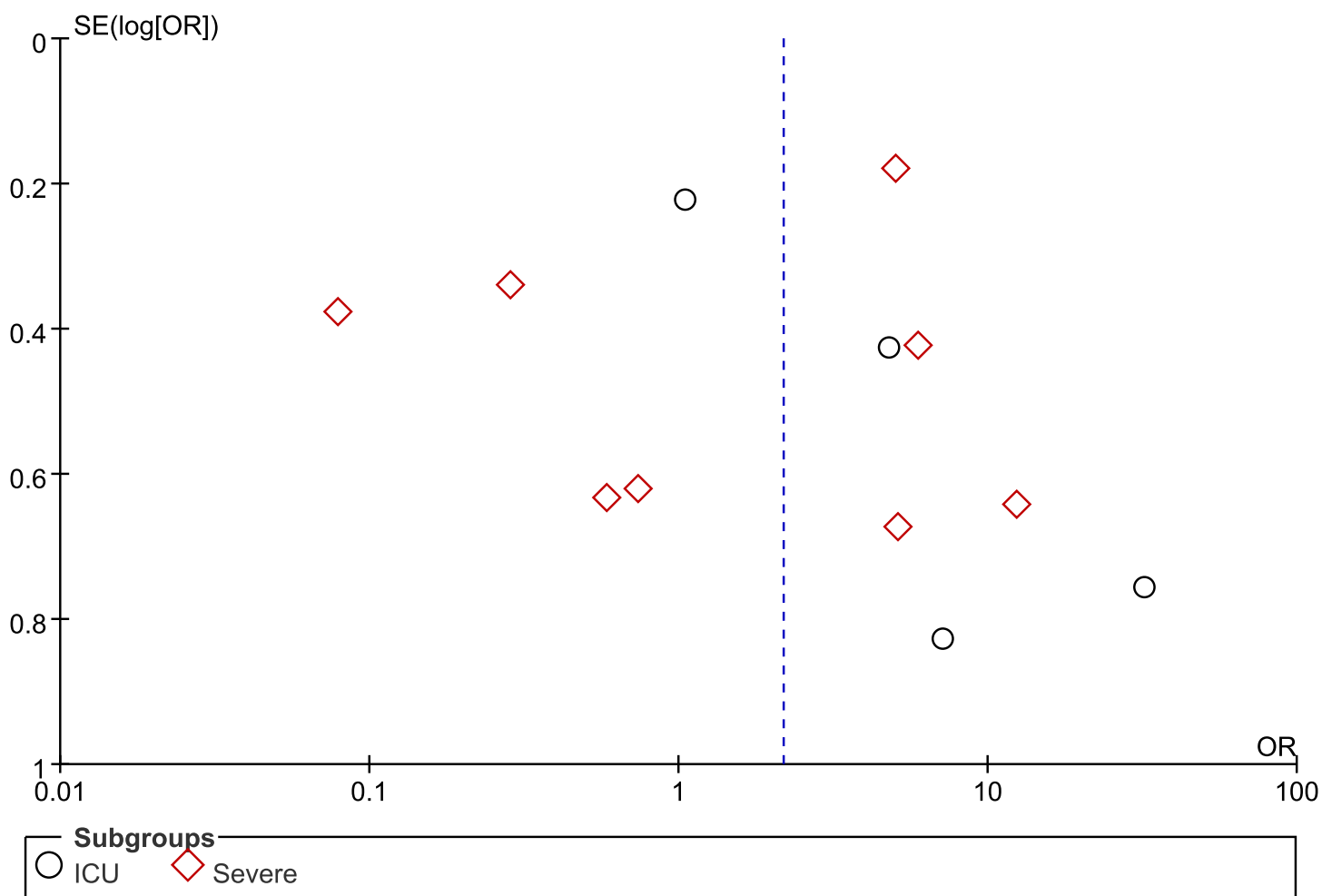

**C**

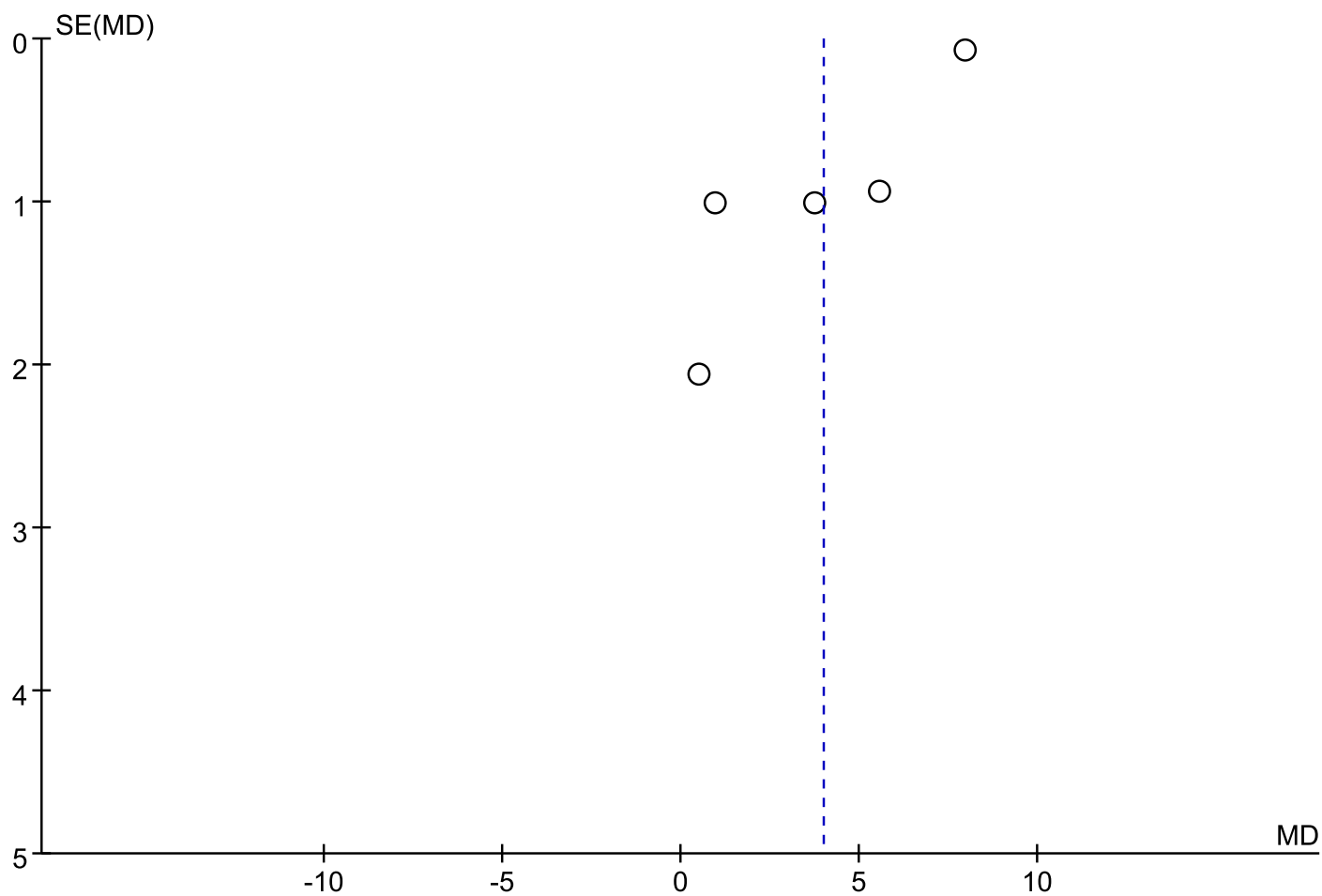

**D**

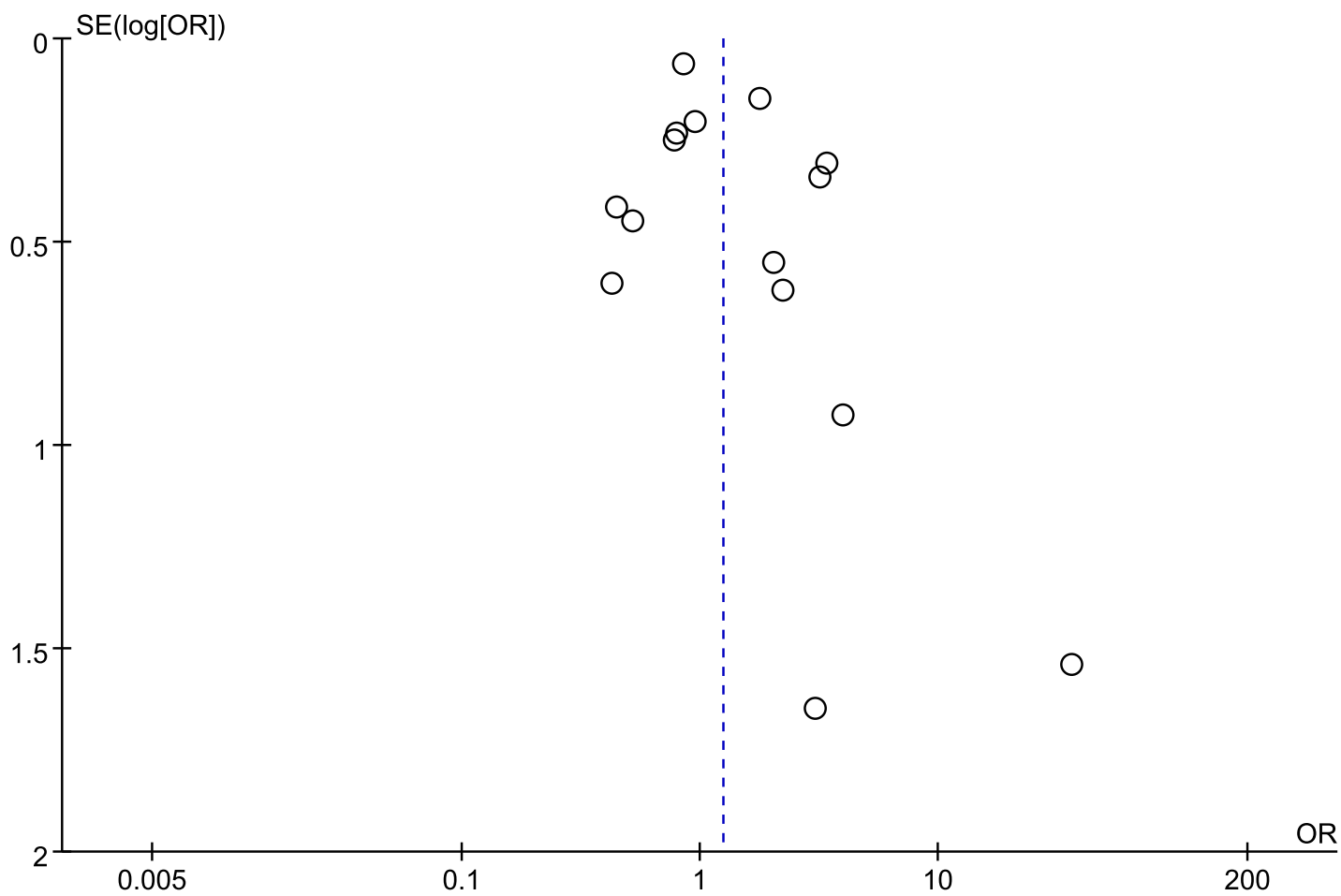

Supplement: S1 Fig — A. Funnel plot of the proportion of corticosteroid treatments in COVID-19 patients by region. B. Funnel plot of the proportion of corticosteroid treatments in COVID-19 patients by severity. C. Funnel plot of the effect of corticosteroid treatments on viral clearance in COVID-19 patients. D. Funnel plot of mortality. (PDF) [file pone.0249481.s001.pdf]

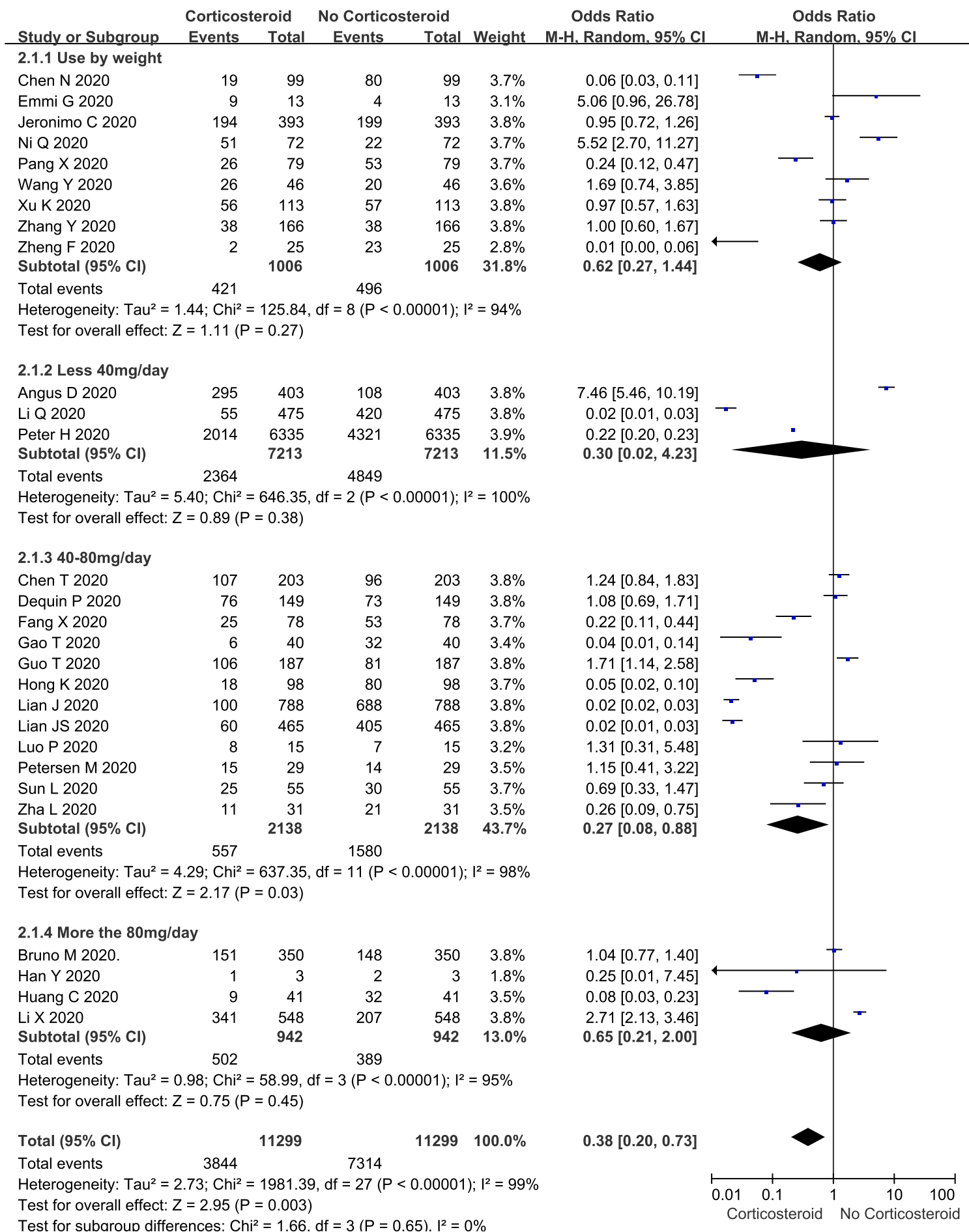

Supplement: S2 Fig — (PDF) [file pone.0249481.s002.pdf]

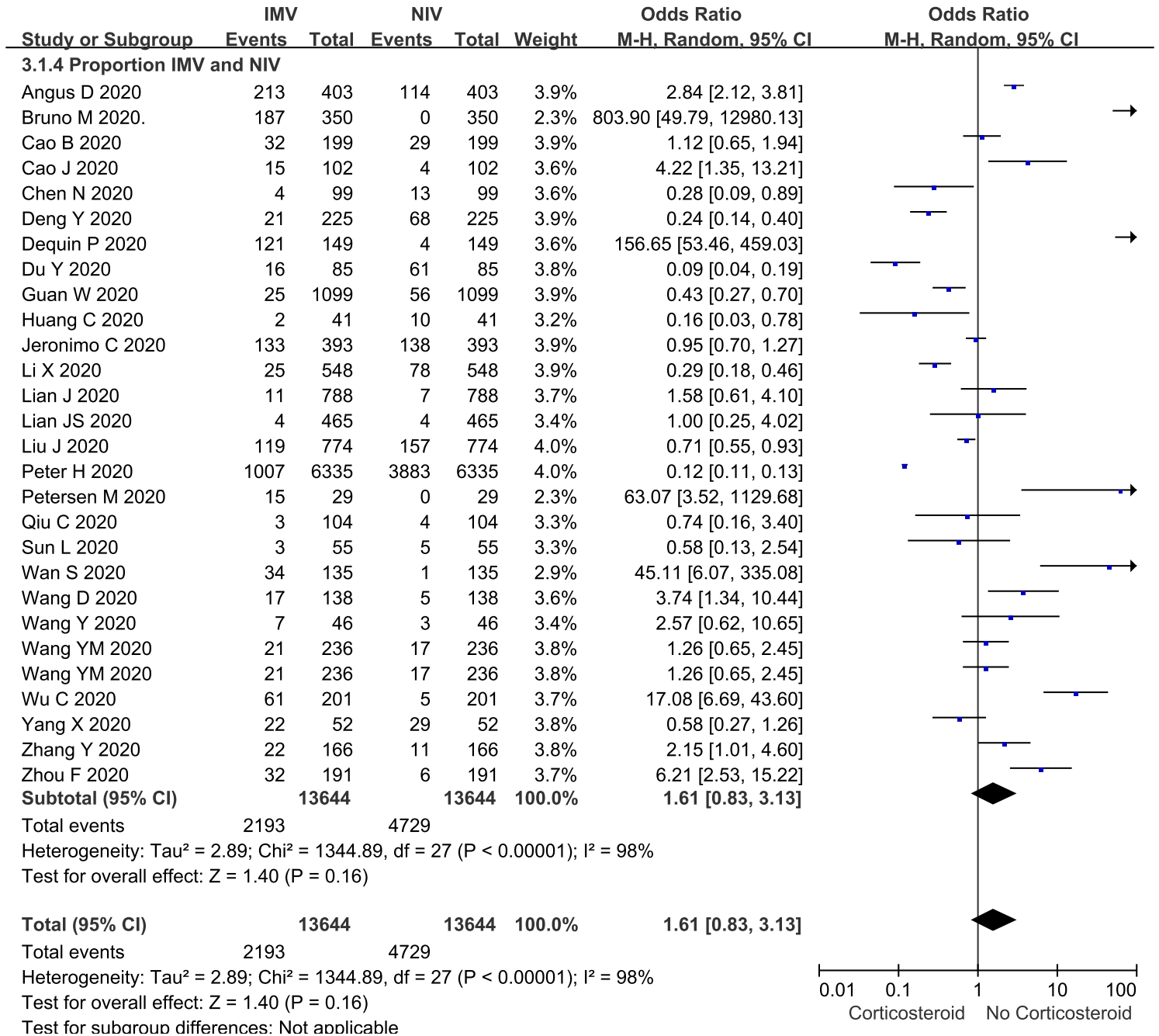

Supplement: S3 Fig — (PDF) [file pone.0249481.s003.pdf]

A

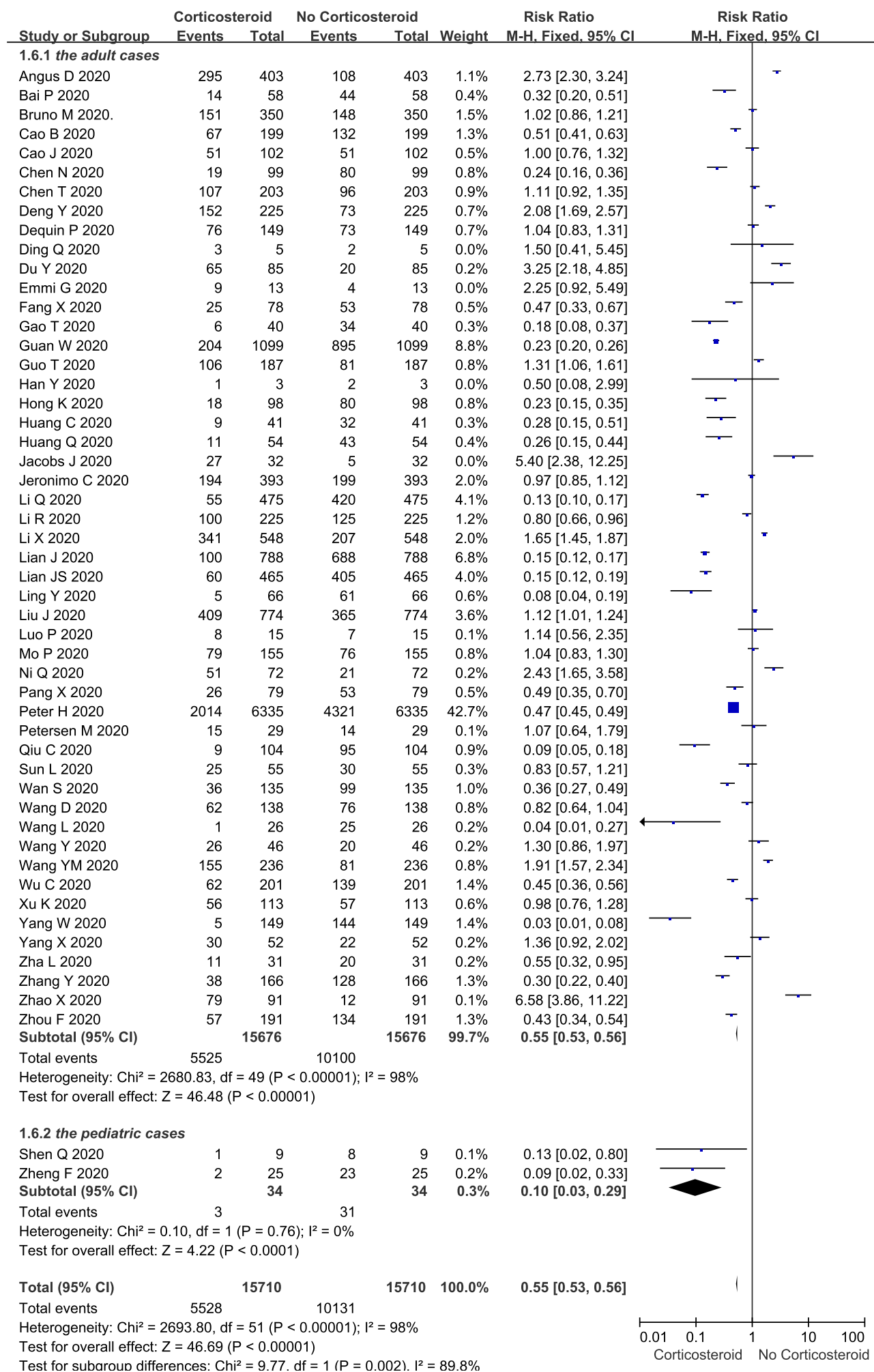

B

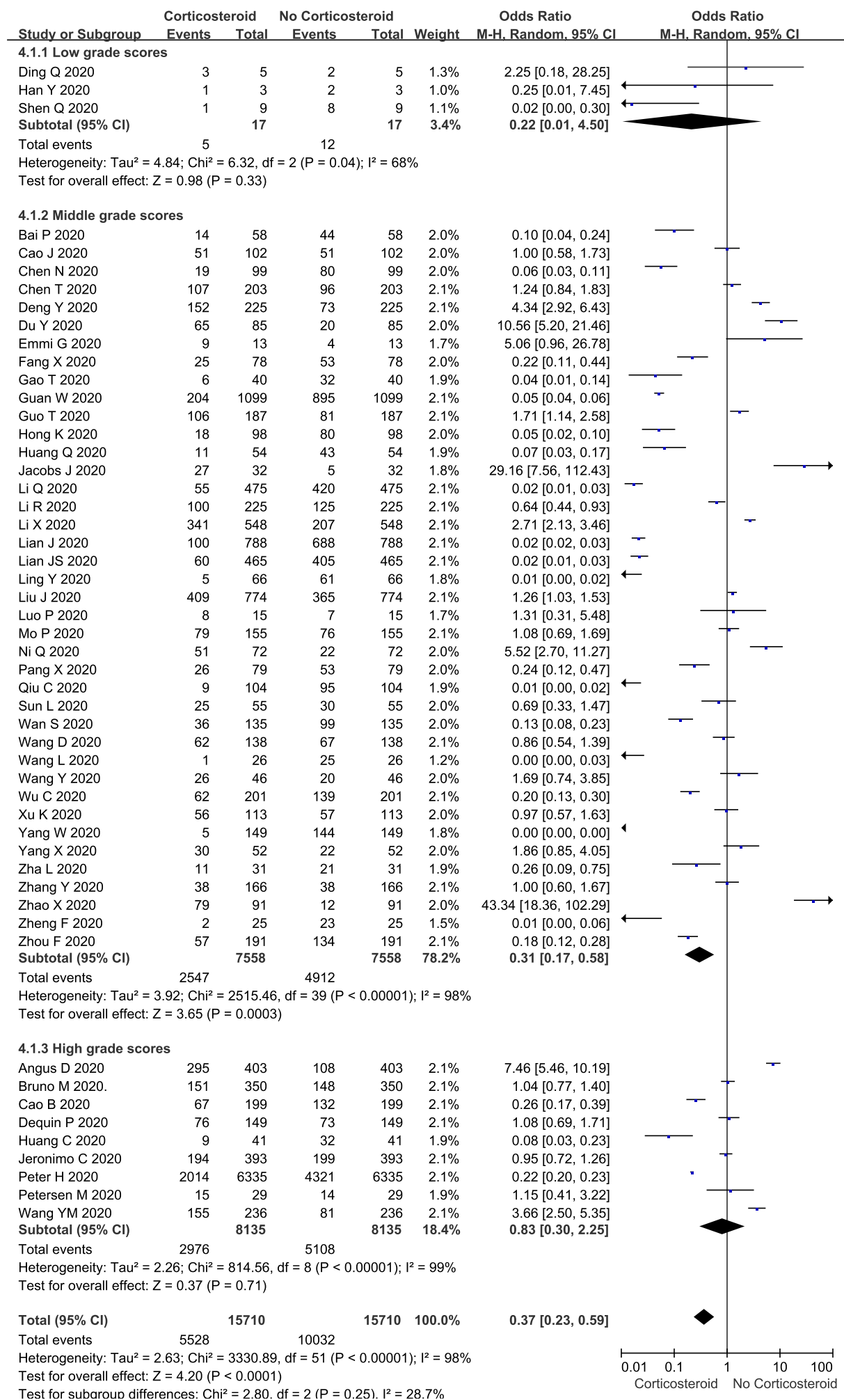

Supplement: S4 Fig — (PDF) [file pone.0249481.s004.pdf]

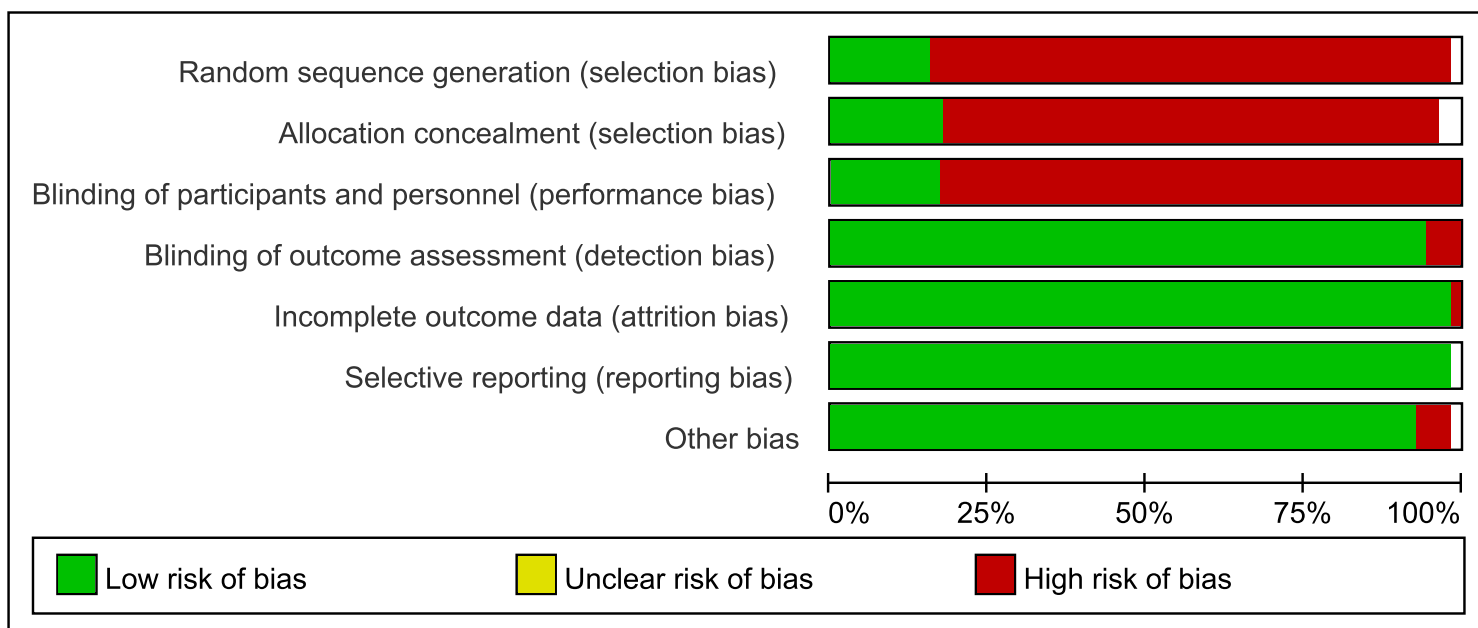

Supplement: S5 Fig — (PDF) [file pone.0249481.s005.pdf]
