# Supplementary material for: The proportion and effect of corticosteroid therapy in patients with COVID-19 infection: A systematic review and meta-analysis
Source: PLoS One. 2021 Apr 21;16(4):e0249481. doi: 10.1371/journal.pone.0249481 (PMC8059814; doi:10.1371/journal.pone.0249481)
Supplement: S6 Fig — (PDF) [file pone.0249481.s006.pdf]

	Random sequence generation (selection bias)	Allocation concealment (selection bias)	Binding of participants and personnel (performance bias)	Binding of outcome assessment (detection bias)	Incomplete outcome data (attrition bias)	Selective reporting (reporting bias)	Other bias
Angus D 2020	+	+	+	+	+	+	+
Bai P 2020	−	−	−	−	+	+	+
Bruno M 2020.	+	+	+	+	+	+	+
Cao B 2020	+	+	+	+	+	+	+
Cao J 2020	−	−	−	+	+	+	+
Chen N 2020	−	−	−	+	+	+	+
Chen T 2020	−	−	−	+	+	+	+
Deng Y 2020	−	−	−	+	+	+	+
Dequin P 2020	+	+	+	+	+	+	+
Ding Q 2020	−	−	−	+	−	+	−
Du Y 2020	−	−	−	+	+	+	+
Emmi G 2020	−	−	−	+	+	+	+
Fang X 2020	−	−	−	+	+	+	+
Gao T 2020	−	−	−	+	+	+	+
Guan W 2020	−	−	−	+	+	+	+
Guo T 2020	−	−	−	+	+	+	+
Han Y 2020	−	−	−	+	+	+	−
Hong K 2020	−	−	−	+	+	+	+
Huang C 2020		+	+	+	+	+	+
Huang Q 2020	−	−	−	+	+	+	+
Jacobs J 2020	−	−	−	+	+	+	+
Jeronimo C 2020	+	+	+	+	+	+	+
Lian J 2020	−	−	−	+	+	+	+
Lian JS 2020	−	−	−	+	+	+	+
Ling Y 2020	−	−	−	+	+	+	+
Li Q 2020	−		−	−	+	+	
Li R 2020	−	−	−	+	+	+	+
Liu J 2020	−		−	−	+		+
Li X 2020	−	−	−	+	+	+	+
Luo P 2020	−	−	−	+	+	+	+
Mo P 2020	−	−	−	+	+	+	+
Ni Q 2020	−	−	−	+	+	+	+
Pang X 2020	−	−	−	+	+	+	+
Petersen M 2020	+	+	+	+	+	+	+
Peter W 2020	+	+	+	+	+	+	+
Qiu C 2020	−	−	−	+	+	+	+
Shen Q 2020	−	−	−	+	+	+	−
Sun L 2020	−	−	−	+	+	+	+
Wang D 2020	−	−	−	+	+	+	+
Wang L 2020	−	−	−	+	+	+	+
Wang Y 2020	−	−	−	+	+	+	+
Wang YM 2020	+	+	+	+	+	+	+
Wan S 2020	−	−	−	+	+	+	+
Wu C 2020	−	−	−	+	+	+	+
Xu K 2020	−	−	−	+	+	+	+
Yang W 2020	−	−	−	+	+	+	+
Yang X 2020	−	−	−	+	+	+	+
Zha L 2020	−	−	−	+	+	+	+
Zhang Y 2020	−	−	−	+	+	+	+
Zhao X 2020	−	−	−	+	+	+	+
Zheng F 2020	−	−	−	+	+	+	+
Zhou F 2020	−	−	−	+	+	+	+

Red plus high risk Yellow plus low risk White blank unclear
