# Supplementary material for: The proportion and effect of corticosteroid therapy in patients with COVID-19 infection: A systematic review and meta-analysis
Source: PLoS One. 2021 Apr 21;16(4):e0249481. doi: 10.1371/journal.pone.0249481 (PMC8059814; doi:10.1371/journal.pone.0249481)
Supplement: S1 Table — (DOC) [file pone.0249481.s007.doc]

| **Section/topic** | **#** | **Checklist item** | **Reported on page #** |
| --- | --- | --- | --- |
| **TITLE** | | |  |
| Title | 1 | The proportion and effect of corticosteroid therapy for patients with COVID-19 infection: a systematic review and meta-analysis | 1 |
| **ABSTRACT** | | |  |
| Structured summary | 2 | Objectives: COVID-19 remains a global challenge. Corticosteroids are a group of anti-inflammatory and suppressive immune response drugs that are widely used in the treatment of COVID-19. Comprehensive reviews investigating the comparative proportion and efficacy of corticosteroid use are scarce. Therefore, we conducted a systematic review and meta-analysis of clinical trials to evaluate the proportion and efficacy of corticosteroid use for the treatment of COVID-19.  Methods: We conducted a comprehensive literature review and meta-analysis of PubMed, EMBASE, the Cochrane Controlled Trials Registry, and the China Academic Journal Network Publishing Database for research articles, including observational studies and clinical trials. Patients from December 1st 2019 to January 1st, 2021. Outcome measures were the proportion of patients administered corticosteroids, viral clearance and mortality. Effect size was reported as weighted mean differences (WMDs) for continuous outcomes and odds ratios (ORs) for dichotomous outcomes with associated 95% confidence intervals (CIs).  Results: Fifty-two trials involving 15710 patients were included. The meta-analysis demonstrated that the proportion of COVID-19 patients who received corticosteroids was significantly lower than that of patients who did not receive corticosteroids (35.19% vs. 64.49%). In addition, our meta-analysis demonstrated no significant difference in the proportions of severe and nonsevere patients who were administered corticosteroids (27.91% vs. 20.91%). We also performed subgroup analyses stratified by whether in ICU, indicating that the proportion of patients administered corticosteroids was significantly higher among intensive care unit (ICU) patients than among non-ICU patients. The results of our meta-analysis indicated that corticosteroid treatment significantly delayed the viral clearance time. Finally, our meta-analysis demonstrated no significant difference between the use of corticosteroids for COVID-19 patients who died and those who survived. This result indicated that mortality was not correlated with corticosteroid therapy.  Conclusion: The proportion of COVID-19 patients who received corticosteroids was significantly lower than that of patients who did not receive corticosteroids. Corticosteroid use in subjects with severe acute respiratory syndrome coronavirus 2 (SARS-CoV-2) infections delayed virus clearance and did not convincingly improve survival; therefore, corticosteroids should be used with extreme caution in the treatment of COVID-19. | 2-3 |
| **INTRODUCTION** | | |  |
| Rationale | 3 | Coronavirus disease 2019 (COVID-19) is a novel viral respiratory disease that occurred in December 2019 and is caused by SARS-CoV-2, a novel, highly diverse, enveloped, positive single-stranded betacoronavirus that belongs to the subgenus Sarbecovirus[1]. The rapid progression of the COVID-19 pandemic has become a global concern. By Central European Time March 11 2020, 114 countries were involved, a total of 118319 laboratory-confirmed infections have been reported, and over 4000 deaths have occurred; the World Health Organization (WHO) declared the COVID-19 outbreak a global pandemic[2]. By June 15, 2020, approximately 7823289 laboratory-confirmed cases have been identified worldwide, with 431541 deaths. Worryingly, the number of newly diagnosed patients continues to rise dramatically [3].  COVID-19 shows clinical manifestations in humans resembling that of viral pneumonia [4]. The pathogenesis of viral pneumonia may not be virus-induced cytopathy but rather an aberrant host immune reaction (e.g., cytokine storm) to the viral infection in all affected patients [5]. Because the immune pathogenesis of pneumonia may be the same in all infected patients, the timing of immunomodulator (corticosteroid) treatment is crucial, and the early control of initial immune-mediated lung injury is helpful for reducing patient morbidity and possibly mortality [5]. Corticosteroids do not directly inhibit virus replication, and their main role is anti-inflammation and suppression of the immune response [6] .  A wide range of variability in COVID-19 severity has been observed, ranging from asymptomatic to critical, and symptoms of the disease are non-specific, including self-reported fever, dry cough, fatigue, and myalgia with diarrhea. Severe cases with difficulty breathing, sepsis, and septic shock have been reported, progressing to a severe form of pneumonia in 10-15% of patients. Severe COVID-19 can lead to critical illness, with acute respiratory distress syndrome (ARDS) and multiorgan failure (MOF) as its primary complications and even fatal respiratory diseases [7]. Its epidemiological and clinical characteristics are slowly becoming evident. However, the pathogenesis feature of acute lung injury in COVID-19 and other infectious respiratory diseases remains unknown. Given the rapid emergence of COVID-19, there are currently no pharmacological therapies of proven efficacy to treat this fatal disease [8]. Several companies have produced vaccines, but they are in phase 2 or 3 clinical trials, and the exact effect of these vaccines remains to be observed in the future [9].SARS-CoV-2, severe acute respiratory syndrome coronavirus (SARS-CoV), and Middle East respiratory syndrome coronavirus (MERS-CoV) share many genetic features, and particularly, SARS-CoV-2 is highly homologous to SARS-CoV [10]. The phylogenetics and clinical features of COVID-19 resemble those of SARS and MERS; however, corticosteroid therapy in the latter two infections is controversial [11, 12]. The current guidance from the WHO on the clinical management of severe acute respiratory infection,when SARS-CoV-2 infection is suspected (released: September 2, 2020), advises that systemic corticosteroids rather than no corticosteroids for the treatment of patients with severe and critical COVID-19, however, suggest not to use corticosteroids in the treatment of patients with non-severe COVID-19 [13, 14].Besides, Dexamethasone, a corticosteroid, has been found to improve survival in hospitalized patients who require supplemental oxygen, with the greatest effect observed in patients who require mechanical ventilation. Therefore, the use of dexamethasone is strongly recommended in this setting by the COVID-19 treatment Guidelines of National Institutes of Health (last update November 3, 2020) [15]. There are several reports on the use of corticosteroids in addition to other therapeutics for patients with COVID-19, especially for persons with severe infection hospitalized in the ICU; their impact on clinical outcomes remains highly controversial [8, 16, 17]. However, there are few data on the proportion and efficacy of corticosteroids in this setting by now [18, 19]. Understanding the evidence for the efficacy and safety of corticosteroid treatment for COVID-19 is of immediate clinical importance. This meta-analysis proposes to evaluate the proportion and effectiveness of the current options for the use of systemic corticosteroid therapy for COVID-19.ence for the efficacy and safety of corticosteroid treatment for COVID-19 is of immediate clinical importance. | 4-6 |
| Objectives | 4 | To evaluate the proportion and effectiveness of the current options for the use of systemic corticosteroid therapy for COVID-19. | 6 |
| **METHODS** | | |  |
| Protocol and registration | 5 | None |  |
| Eligibility criteria | 6 | Inclusion criteria were as follows: 1) research articles, including observational studies and clinical trials, on the use of glucocorticoids in persons with COVID-19 infection, who were diagnosed by real-time reverse transcription-polymerase chain reaction (RT-PCR) and underwent chest X-rays or chest computed tomography (CT), in hospitalization; (2) reported outcomes on the proportion of glucocorticoids administered by the severity and regionCOVID-19, virus clearance and/or death. (3) There was no restriction by country in which the trial occurred and age.  The exclusion criteria were as follows: 1) post-transplant or any organ transplant recipient patients; 2) studies that did not report original data, clear diagnostic criteria or data that could be summarized as the mean and standard deviation or that had no reliable clinical data; and 3) conference abstracts, or review articles.  Disagreements about study selections were resolved by discussion with a review author (YK Wang) until consensus was reached. | 7 |
| Information sources | 7 | PubMed, Embase, the Cochrane Controlled Trials Registry, and the China Academic Journal Network Publishing Database from December 1st 2019 to January1st, 2021 | 7 |
| Search | 8 | the following key words: glucocorticoid or corticosteroid or adrenal cortex hormones or steroid or corticoid or corticoids or corticosteroids or glucocorticosteroid or glucocorticosteroids or methylprednisolone or budesonide or dexamethasone or Prednisone or prednisolone or methylprednisolone or hydrocortisone or cortisol. Each of these was searched with the following string of key words (using the “AND” operator): COVID-19 OR coronavirus OR "SARS-CoV-2" OR "novel coronavirus" OR 2019-nCoV OR "Severe Acute Respiratory Syndrome Coronavirus 2" OR "Corona Virus Disease 2019" OR COVID-19 OR COVID. No language was restricted to search published studies. | 7 |
| Study selection | 9 | I Inclusion criteria were as follows: 1) research articles, including observational studies and clinical trials, on the use of glucocorticoids in persons with COVID-19 infection, who were diagnosed by real-time reverse transcription-polymerase chain reaction (RT-PCR) and underwent chest X-rays or chest computed tomography (CT), in hospitalization; (2) reported outcomes on the proportion of glucocorticoids administered by the severity and regionCOVID-19, virus clearance and/or death. (3) There was no restriction by country in which the trial occurred and age.  The exclusion criteria were as follows: 1) post-transplant or any organ transplant recipient patients; 2) studies that did not report original data, clear diagnostic criteria or data that could be summarized as the mean and standard deviation or that had no reliable clinical data; and 3) conference abstracts, or review articles.  Disagreements about study selections were resolved by discussion with a review author (YK Wang) until consensus was reached. | 7 |
| Data collection process | 10 | Two researchers (Yao Lu, J-N Wang) independently performed data extraction. Means were obtained from data tables or figures if no direct data were available from the article text or the corresponding author. | 7 |
| Data items | 11 | If the sample mean and standard deviation of the data could not be obtained from the authors, they were calculated from the sample size, median, range and/or interquartile range according to the procedures in articles by Wan X and Luo D et al[18, 19]. Disagreements about data extraction were resolved by discussion with a review author (Y-K Wang) until consensus was reached. | 7 |
| Risk of bias in individual studies | 12 | Two researchers (P-WChen, J-B Guo) independently assessed the quality of the included studies. The risk of bias was evaluated using the modified Jadad scale [20]. Categories included: “Was the study described as randomized?”, “Was the method used to generate the sequence of randomization described and appropriate (random numbers, computer-generated, etc.)?”, “Was the study described as double-blind?”, “Was the method of double-blinding described and appropriate (identical placebo, active placebo, dummy, etc.)?”, and “Was there a description of withdrawals and drop-outs?”. The Jadad scale is a five-point scale; a score of zero indicates poor quality evidence, and a score of five indicates high quality evidence; therefore, trials with a score of 4 or 5 were considered to be of high methodological quality. Disagreements about study quality were resolved by discussion with a review author (Y-K Wang) until consensus was reached. | 8-9 |
| Summary measures | 13 | Data were analyzed using the Cochrane Collaboration software Review Manager 5.3. Weighted mean differences (WMDs) and their associated 95% confidence intervals (CIs) were calculated for the continuous outcome of virus clearance, while odds ratios (ORs) and their associated 95% CIs were calculated for the dichotomous outcomes, proportion of glucocorticoids used and mortality. | 9 |
| Synthesis of results | 14 | Heterogeneity was assessed using the I2-test. A fixed-effects model was used to pool data if there was no evidence of significant heterogeneity (I2≤50%). Otherwise, a random-effects model was used. | 9 |

Page 1 of 2

| **Section/topic** | **#** | **Checklist item** | **Reported on page #** |
| --- | --- | --- | --- |
| Risk of bias across studies | 15 | Publication bias was assessed with funnel plots. | 9 |
| Additional analyses | 16 | Subgroup analyses were stratified by area (Wuhan, China; Outside of Wuhan, China; and Outside of China), severity (ICU and severe), evidence grade age (pediatric or adult) and the dosage of glucocorticoids used. | 9 |
| **RESULTS** | | |  |
| Study selection | 17 | The searches identified 2326 relevant articles. Of these, 52 trials were eligible for inclusion according to our criteria for considering studies for this meta-analysis [18, 19, 23-72] (Figure 1). Forty-four trials were retrospective case series (RCS), and eight trials were randomized controlled trials (RCTs). There are 11 RCT protocols which were not included, still no results (S2 Table). A total of 15710 patients with COVID-19 were included in the analyses. | 10 |
| Study characteristics | 18 | Among the 52 included trials, 18 were multicenter trials, and 35 were single-center trials. Twenty-six trials were conducted in Wuhan, China, 17 were conducted outside of Wuhan, China, and the 9 trials were conducted outside of China. 12 studies performed analyses by severity; 4 trials divided patients into the ICU or non-ICU groups, and 8 trials divided patients into the severe or nonsevere groups. Viral clearance was compared in 5 trials. The effect of mortality was analyzed in 15 trials. Most of the trials indicated that 40-80 mg methylprednisolone was used once or twice per day, ranging from 4-15 days. Antibiotics were not administered in three trials, 1 trial had no antibiotics data, and 51 trials administered antibiotics. 2193 patients used NIV, 4729 patients of 27 trials used IMV to assist ventilation (S3 Fig). The 80 patients of 14 trials were treated with ECMO. 32 patients are included in the Jacobs J, et al’ article showed that COVID-19 patients with severe pulmonary compromise supported with ECMO may play a useful role in salvaging select critically patients [29].The most common complications were ARDS, acute coagulopathy, acute liver injury and acute kidney injury. The characteristics of the 52 included trials are summarized in Table 1. | 10 |
| Risk of bias within studies | 19 | The level of evidence for each trial was graded from 1 to 5 according to the Jadad quality score (Table 1 and S3 Table). For publication bias, the shape of the funnel plot showed obvious asymmetry for trials investigating the proportion of corticosteroid use for COVID-19 patients, regardless of region or severity (S1 Fig A,B), but slight asymmetry for trials investigating the effect of viral clearance (S1 Fig C) and mortality (S1 Fig D).  Besides, the risk of bias, as assessed by the Cochrane tool, is summarized in S5 Fig and presented in detail in S6 Fig. The mainly limitation of the included trials was selection bias and performance bias, because most of the studies were not random and blinding researches. | 13-14 |
| Results of individual studies | 20 | The proportion of corticosteroid treatments  The proportion of COVID-19 patients treated with corticosteroids vs. those that were not is described in all 52 included trials (n=15710 patients). The meta-analysis demonstrated that the proportion of COVID-19 patients treated with corticosteroids was significantly lower than that of patients who were not treated with corticosteroids (35.19% vs. 64.49%, 5528 vs. 10131 OR: 0.35, 95% CI: 0.22-0.56, P <0.01; Figure 2), no matter adult or pediatric cases (S4A Fig). There was evidence of significant heterogeneity between trials (P <0.01, I2 = 98%). There was no significant difference between the patients who were treated with corticosteroids and without corticosteroids in the low and high jadad scores (S4B Fig).  Figure 2 Proportions of corticosteroid administration in COVID-19 patients: Overall and subgroup analyses stratified by region.  Comparing the proportion of severe patients to the proportion of nonsevere patients administered corticosteroids  The proportion of severe patients treated with corticosteroids was 32.05% (n=317) vs. 22.31% (n=445) of nonsevere patients treated with corticosteroids in 12 trials (n=2983 patients). Our meta-analysis demonstrated there was significant difference in the proportions of severe plus ICU and nonsevere plus no ICU patients administered corticosteroids (OR: 2.17, 95% CI: 0.86-5.46, P =0.04; Figure 3). There was evidence of significant heterogeneity between trials (P <0.01, I2 = 94%).  Figure 3 Proportions of severe and nonsevere patients administered corticosteroids: Overall and subgroup analyses stratified by severity.  The effect of corticosteroid use on viral clearance  We evaluated the viral clearance time in patients treated with corticosteroids and compared it to that of patients treated without corticosteroids using a random-effects model (Figure 4).There five studies have the outcome of viral clearance. All of the 5 studies viral clearance was confirmed by serial RT-PCR of samples from throat swabs or sputum; clearance was defined as having at least two consecutive negative results in the four studies. The pooled estimates showed that corticosteroid treatment significantly delayed the viral clearance time (WMD: 3.98, 95% CI: 0.76-7.02, P < 0.05; I2 =95%). However, there was significant heterogeneity between studies.  Figure 4 Corticosteroid vs. non-corticosteroid treatment: viral clearance time (days).  The effect of corticosteroid use on mortality  The mortality of COVID-19 patients treated with corticosteroids for 4-15 days was described in 15 trials (n=9279 patients). The meta-analysis demonstrated no significant difference in the use of corticosteroids between COVID-19 patients who died and those who survived (overall OR: 1.24, 95% CI 0.89-1.73, P=0.2; Figure 5). There was evidence of significant heterogeneity between trials (P < 0.01, I2 = 80%).  Figure 5 Corticosteroid vs. non-corticosteroid treatment: mortality of studied subjects (both of the two groups received the corticosteroid).non-corticosteroid treatment: mortality of studied subjects (both of the two groups received the corticosteroid). | 10-12 |
| Synthesis of results | 21 | The proportion of COVID-19 patients administered corticosteroids was significantly lower than that of patients who were not administered corticosteroids. Subgroup analyses stratified by severity indicated that the proportion of corticosteroid use was significantly higher in ICU patients than in non-ICU patients. Corticosteroid use in subjects with SARS-CoV-2 infection resulted in delay virus clearing and did not convincingly improve survival. Therefore, corticosteroids should be used with extreme caution in the treatment of COVID-19. Nevertheless, further multicenter，larger randomized, controlled clinical trials are needed to verify this conclusion. | 18 |
| Risk of bias across studies | 22 | The level of evidence for each trial was graded from 1 to 5 according to the Jadad quality score (Table 1 and S3 Table). For publication bias, the shape of the funnel plot showed obvious asymmetry for trials investigating the proportion of corticosteroid use for COVID-19 patients, regardless of region or severity (S1 Fig A,B), but slight asymmetry for trials investigating the effect of viral clearance (S1 Fig C) and mortality (S1 Fig D).  Besides, the risk of bias, as assessed by the Cochrane tool, is summarized in S5 Fig and presented in detail in S6 Fig. The mainly limitation of the included trials was selection bias and performance bias, because most of the studies were not random and blinding researches. | 13-14 |
| Additional analysis | 23 | Subgroup analyses stratified by region indicated that the proportion of COVID-19 patients administered corticosteroids was significantly lower than that of patients who were not administered corticosteroids in Wuhan, China (OR: 0.40, 95% CI: 0.20-0.82, P =0.01; I2 = 98%, Figure 2) and outside of Wuhan (OR: 0.13, 95% CI: 0.04-0.38, P < 0.01; I2 =99%, Figure 2),but there are no significantly difference in outside of China (OR: 1.26, 95% CI: 0.43-3.64, P =0.68; I2 = 99%, Figure 2).  Subgroup analyses were also stratified by whether in ICU and severity. Patients who were identified as severe or critical were collectively considered the “severe” group, while those mild and common COVID-19 patients were considered the “nonsevere” group. The subgroup analysis indicated that the proportion of patients treated with corticosteroids was significantly higher among ICU patients than among non-ICU patients (OR: 5.19 95% CI: 1.21, 22.14 P =0.03; I2 = 89%; Figure 3), but there was no significant difference in the proportion of critical and severe patients versus mild and common level patients treated with corticosteroids (OR: 1.42 95% CI: 0. 37-5.41 P =0.61; I2 = 96%; Figure 3).  Subgroup analyses were also stratified by dosage of corticosteroids, ventilated and non-ventilated patients. The dosage of corticosteroids used mainly 40-80mg/day. The proportion of patients treated with corticosteroids 40-80 mg/day was significantly lower than without corticosteroid patients (the patients’ number 557 vs.1580; S2 Fig), but there was no significant difference in the proportion of use by weight, less 40mg/day and more than 80mg/day group. There was also no significant difference ventilated and non-ventilated patients (the patients’ number 2193vs.4729; S3 Fig). | 12-13 |
| **DISCUSSION** | | |  |
| Summary of evidence | 24 | Since the outbreak of the novel SARS-CoV-2 infection, no effective antiviral treatment has been developed. COVID-19 patients are mainly treated with symptomatic therapy. In clinical practice, corticosteroids are widely used in the symptomatic treatment of severe viral pneumonia. However, there has been a lot of controversy as to whether COVID-19 patients should be adjunctive treated with corticosteroids. The pathological feature of COVID-19 pneumonia is an inflammatory reaction by the destruction of the deep airway and alveoli [73]. The current hypothesis is that the lung injury is not associated with direct virus-induced injury but that COVID-19 invasion triggers immune responses and inflammatory response that lead to the activation of immune cells (macrophages, T and B-lymphocytes, granulocytes, monocytes) to release a large number of pro- and anti-inflammatory cytokines, including TNF-α, IL-1β, and IL-6 and markedly increased inflammatory markers such as CRP and ESR [74]. The overwhelming secretion of cytokines causes severe alveolar and deep airway damage, which manifests as extensive damage to pulmonary vascular endothelial and alveolar epithelial cells as well as increased pulmonary vascular permeability, resulting in pulmonary edema and hyaline membrane formation [75]. Lung histologic examination has shown diffuse alveolar damage with cellular fibromyxoid exudates and hyaline membrane formation, which is similar to acute respiratory distress syndrome [73]. Further autopsy has revealed bilateral diffuse alveolar injury with fibrous mucinous exudate and interstitial mononuclear inflammatory infiltration dominated by lymphocytes, which is very similar to SARS-CoV and MERS-CoV infections [73]. This indicates that patients with COVID-19 are usually accompanied by increased immune factors and inflammatory responses, and the concentrations of immune factors are associated with the severity of the disease [62]. Corticosteroids are classical immunosuppressive drugs that perform key physiological processes, including inhibitory effects on the immune response and anti-inflammatory roles to reduce systemic inflammation [16, 76]. Both are important in stopping or delaying the progression of pneumonia. Low-dose corticosteroids have been proven to be effective in the treatment of viral pneumonia due to their excellent pharmacological effects on the suppression of the immune system to prevent the development of related autoimmune diseases and dysfunctional systematic inflammation [77].  In this meta-analysis, the proportion of COVID-19 patients administered corticosteroids was significantly lower than that of patients who did not receive corticosteroids. Subgroup analyses stratified by region showed that the proportion of COVID-19 patients administered corticosteroids was significantly lower than that of patients who were not in Wuhan, China, outside of Wuhan, and outside of China. The results of this study indicate that the clinical application of corticosteroids is not very common. Thus, the use of corticosteroids could be regarded as a double edge sword [16].  Studies have indicated that patients with severe conditions are more likely to require adjunctive corticosteroid therapy [77]. However, our meta-analysis demonstrated no significant difference in the proportion of severe and nonsevere patients administered corticosteroids. This is different view from the results of previous research. We speculate that the reason underlying this inconsistency is an unsuitable population selection: mild or common COVID-19 might not be included as a target population to assess the effectiveness of corticosteroids in most studies. We also performed subgroup analyses stratified by severity, which indicated that the proportion of corticosteroid use was significantly higher in ICU patients than in non-ICU patients. The results indicate that ICU patients were more likely to require corticosteroid therapy. The meta-analysis from Li Huan. et al. reported that evidence suggests that ICU inpatients with coronavirus infections were more likely to receive corticosteroids than non-ICU inpatients[78].  The results of our meta-analysis indicate that corticosteroid treatment significantly delayed the viral clearance time. The study by Russell D.C. et al showed a delay in viral RNA clearance from the respiratory tract and suggested that this followed corticosteroid administration for a MERS-CoV infection [14]. Moreover, a prospective, randomized double-blinded, placebo-controlled trial on SARS, compared early adjunctive hydrocortisone treatment (before day seven of the illness) with a placebo and showed that early adjunctive hydrocortisone therapy in patients was associated with delayed SARS-CoV RNA clearance in plasma [79].  The meta-analysis demonstrated no significant difference in the use of corticosteroids between COVID-19 patients who died and those who survived. The results indicate that mortality was not correlated with corticosteroid therapy; there was no favorable impact on the endpoint of death. In a retrospective cohort study reporting 309 patients who were critically ill with MERS [12], the authors reported that administration of corticosteroids compared to non-corticosteroids group no difference in 90-day mortality, but was associated with delayed MERS-CoV RNA clearance from respiratory tract secretions. This finding was somewhat confirmed in our systematic review. Glucocorticoid therapy was associated with delayed SARS-CoV-2 RNA clearance after adjustment for baseline and time-varying confounding factors [33]. However, the WHO rapid evidence appraisal for covid-19 therapies working group prospective meta-analysis show that clinical trials of critically ill patients with COVID-19, administration of systemic corticosteroids, compared with usual care or placebo, was associated with lower 28-day all-cause mortality [80].That is the different from our result, because we included the mild, common and the severe cases in our meta-analysis. | 16-17 |
| Limitations | 25 | There are some limitations in this meta-analysis. First, some of the included studies were early retrospective cohort studies with small patient sample sizes, historical control studies on this emerging pathogen, and we found substantial heterogeneity between studies, with a low level of evidence that restricted the quality grade of the effects. Larger-scale RCTs are urgently needed. Second, there is no uniform standard for the dosage and initial time of administration of the corticosteroid regimens used in the different studies. For instance, in future research, corticosteroids should be used at the early stage of the illness. Third, antiviral agents might be confounders to corticosteroid use and their effects. Other co-treatments might have influenced our results. Fourthly, our study was not registered, and that study populations include only hospitalized patients. Finally, due to the ongoing outbreak of COVID-19, many regions affected by COVID-19 have not published results on their populations, which may lead to publication bias. | 17 |
| Conclusions | 26 | The proportion of COVID-19 patients administered corticosteroids was significantly lower than that of patients who were not administered corticosteroids. Subgroup analyses stratified by severity indicated that the proportion of corticosteroid use was significantly higher in ICU patients than in non-ICU patients. Corticosteroid use in subjects with SARS-CoV-2 infection resulted in delay virus clearing and did not convincingly improve survival. Therefore, corticosteroids should be used with extreme caution in the treatment of COVID-19. Nevertheless, further multicenter，larger randomized, controlled clinical trials are needed to verify this conclusion. | 17 |
| **FUNDING** | | |  |
| Funding | 27 | This work was supported by the Shaanxi Natural Science Foundation of China (Number 2019JQ-536) |  |

*From:*  Moher D, Liberati A, Tetzlaff J, Altman DG, The PRISMA Group (2009). Preferred Reporting Items for Systematic Reviews and Meta-Analyses: The PRISMA Statement. PLoS Med 6(7): e1000097. doi:10.1371/journal.pmed1000097

For more information, visit: **www.prisma-statement.org**.

Page 2 of 2
