# Supplementary material for: The proportion and effect of corticosteroid therapy in patients with COVID-19 infection: A systematic review and meta-analysis
Source: PLoS One. 2021 Apr 21;16(4):e0249481. doi: 10.1371/journal.pone.0249481 (PMC8059814; doi:10.1371/journal.pone.0249481)
Supplement: S2 Table — (DOCX) [file pone.0249481.s008.docx]

| **Jadad score** | | | |
| --- | --- | --- | --- |
| **Random allocation** | Cited +1 | Described and appropriate +1 | Non-appropriate -1 |
| **Double blind** | Cited +1 | Described and appropriate +1 | Non-appropriate -1 |
| **Dropouts and withdrawals** | Described +1 |  |  |
