# Supplementary material for: The proportion and effect of corticosteroid therapy in patients with COVID-19 infection: A systematic review and meta-analysis
Source: PLoS One. 2021 Apr 21;16(4):e0249481. doi: 10.1371/journal.pone.0249481 (PMC8059814; doi:10.1371/journal.pone.0249481)
Supplement: S1 File — (DOCX) [file pone.0249481.s009.docx]

**11 RCT protocols**

[1] NCT04273321. Efficacy and Safety of Corticosteroids in COVID-19. 2020 : -.

[2] NCT04329650. Efficacy and Safety of Siltuximab vs. Corticosteroids in Hospitalized Patients With COVID-19 Pneumonia. 2020 : -.

[3] NCT04330586. A Trial of Ciclesonide in Adults With Mild COVID-19. 2020 : -.

[4] NCT04341038. Clinical Trial to Evaluate Methylprednisolone Pulses and Tacrolimus in Patients With COVID-19 Lung Injury. 2020 : -.

[5] NCT04343729. Methylprednisolone in the Treatment of Patients With Signs of Severe Acute Respiratory Syndrome in SARS-CoV2: a Randomized, Double-blind, Placebo-controlled Clinical Trial. 2020 : -.

[6] NCT04344288. Corticosteroids During Covid-19 Viral Pneumonia Related to SARS-Cov-2 Infection. 2020 : -.

[7] NCT04344730. Dexamethasone and Oxygen Support Strategies in ICU Patients With Covid-19 Pneumonia. 2020 : -.

[8] NCT04345445. Study to Evaluate the Efficacy and Safety of Tocilizumab Versus Corticosteroids in Hospitalised COVID-19 Patients With High Risk of Progression. 2020 : -.

[9] NCT04347980. Dexamethasone Treatment for Severe Acute Respiratory Distress Syndrome Induced by COVID-19. 2020 : -.

[10] NCT04349410. The Fleming [FMTVDM] Directed CoVid-19 Treatment Protocol. 2020 : -.

[11] ChiCTR2000029386 QY-Y, Zhou Y-H, Lu Y-Q, et al. Effectiveness of glucocorticoid therapy in patients with severe coronavirus disease 2019: protocol of a randomized controlled trial. Chinese medical journalChin. Med. J.. 2020. 133(9): 1080-1086
